# Multilevel meta-analysis of the effect of exercise intervention on inhibitory control in children with ASD

**DOI:** 10.3389/fpsyg.2025.1632555

**Published:** 2025-10-09

**Authors:** Xiangqin Song, Yaoqi Hou, Zonghan Lei, Shenning Zhou

**Affiliations:** ^1^College of Physical Education and Sports, Beijing Normal University, Beijing, China; ^2^University of New South Wales, Sydney, NSW, Australia

**Keywords:** autism spectrum disorder, executive function, physical activity, cognitive enhancement, neurodevelopmental disorders, multilevel meta-analysis

## Abstract

**Background:**

Inhibitory control deficits represent a core cognitive challenge for children with Autism Spectrum Disorder (ASD), impacting behavioral regulation, social interaction, and adaptive functioning. Exercise interventions have emerged as promising non-pharmacological approaches for cognitive enhancement, yet their specific effects on inhibitory control in the ASD population remain unclear. This study aimed to systematically evaluate the efficacy of exercise interventions on inhibitory control in children and adolescents with ASD.

**Methods:**

This systematic review and multilevel meta-analysis followed PRISMA guidelines and was prospectively registered (PROSPERO: CRD420251039964). We systematically searched five electronic databases (PubMed, Web of Science, Embase, Cochrane Library, and CNKI) from inception to December 2023. Randomized and quasi-randomized controlled trials evaluating exercise interventions for inhibitory control in ASD populations were included. Study quality was assessed using the Cochrane Risk of Bias Tool (ROB2). Effect sizes were calculated as standardized mean differences (SMDs) and synthesized using a three-level meta-analytic approach to account for dependency among effect sizes. Subgroup analyses and meta-regression explored potential moderators of intervention efficacy.

**Results:**

Ten studies comprising 466 participants (229 in intervention groups) met inclusion criteria. Exercise interventions demonstrated significant positive effects on inhibitory control in children with ASD (SMD = 0.66, 95% CI [0.44, 0.88]). Structured exercise interventions showed differential efficacy, with Mini Basketball (SMD = 0.95), Martial Arts (SMD = 0.90), and Bicycle Learning (SMD = 0.86) yielding the largest effects. Meta-regression identified total training duration as a significant positive predictor of intervention efficacy (*β* = 0.027, *p* = 0.015), while participant age showed a potential negative association (*β* = −0.091, *p* = 0.083), suggesting enhanced effectiveness in younger children.

**Conclusion:**

This meta-analysis provides robust evidence supporting exercise interventions, particularly structured activities combining physical and cognitive demands, for improving inhibitory control in children with ASD. The findings suggest important clinical implications for intervention design, highlighting the value of adequate intervention duration and early implementation. Future research should address methodological limitations through high-quality trials with standardized protocols and extended follow-up periods.

**Systamatic review:**

CRD420251039964.

## 1 Introduction

Autism Spectrum Disorder (ASD) is a neurodevelopmental disorder characterized by impairments in social communication, repetitive behavior patterns, and restricted interests (Doernberg and Hollander, 2016). In children with ASD, executive function deficits, particularly insufficient inhibitory control, represent a significant core deficit (Schmitt et al., 2018). Inhibitory control refers to an individual’s ability to suppress dominant responses, halt ongoing responses, and control interference, which is crucial for adaptive functioning in daily life (Gligorović and Buha Ðurović, 2014). For children with ASD, deficits in inhibitory control lead to difficulties in behavioral regulation, emotional management problems, and social interaction impairments, subsequently affecting their academic performance, interpersonal relationships, and quality of life (Schmitt et al., 2018). Investigating the manifestation of inhibitory control in autism and its intervention methods holds significant importance. Inhibitory control deficits are closely associated with core ASD symptoms and may serve as an underlying mechanism for various behavioral problems in autism (Kana et al., 2007). Improvements in inhibitory control ability may alleviate a series of functional impairments in individuals with ASD, such as stereotypical behaviors, attention-shifting difficulties, and social adaptation problems (Christ et al., 2007). From a public health perspective, approximately 1/31. According to estimates from the Centers for Disease Control and Prevention’s (CDC) Autism and Developmental Disabilities Monitoring (ADDM) network, 2% of 8-year-old children are diagnosed with ASD (Shaw et al., 2025), imposing an increasing burden on individuals, families, and society, making the search for effective interventions urgent.

Currently, intervention methods for ASD primarily include behavioral therapy, pharmacological treatment, cognitive behavioral therapy, and sensory integration training (Zhuang et al., 2024). Although these methods contribute to improving autism symptoms to some extent, they have numerous limitations in targeting inhibitory control abilities. Pharmacological treatments (such as risperidone and aripiprazole) may temporarily alleviate some symptoms, but their long-term efficacy is limited, and side effects are significant (Choi et al., 2019). Additionally, cognitive behavioral therapy may be effective for high-functioning autism patients but shows poor efficacy for patients with limited cognitive functioning (You et al., 2024).

Compared to traditional intervention methods, exercise intervention, as a non-invasive, low-cost, and easily implemented intervention strategy, has received widespread attention in the field of autism in recent years (Dominguez et al., 2023). Exercise intervention not only improves inhibitory control abilities in children with ASD but also brings multiple health benefits. From a mental health perspective, regular exercise can alleviate anxiety, depressive symptoms, and enhance self-efficacy (Healy et al., 2018). Furthermore, exercise intervention can improve cardiopulmonary function, muscle strength, and coordination, which has positive effects on the motor skill deficits commonly present in children with ASD (Sorensen and Zarrett, 2014). Clinical guidelines across different countries hold varying attitudes toward the application of exercise interventions in autism.

To date, research on the impact of exercise interventions on inhibitory control in children with ASD has primarily focused on specific exercise types (such as aerobic exercise, yoga) or short-term effect assessments, lacking systematic comparisons of different exercise forms, intensities, and durations. Existing research exhibits significant methodological limitations: first, high heterogeneity between studies, with significant differences in intervention design, inhibitory control measurement tools, and statistical analysis methods, resulting in low comparability of results (Hou et al., 2024).

Tan conducted a comprehensive meta-analysis evaluating the effects of physical exercise on cognitive function in individuals with ASD and ADHD. The study analyzed data from 22 studies involving 579 participants aged 3–25 years, with results indicating small to moderate positive effects of exercise on cognition. Specifically regarding inhibitory control, they reported positive effects (*r* = 0.097). However, this study employed traditional random effects models that failed to adequately consider the dependency issue between effect sizes in multi-arm studies. When studies include multiple intervention groups or multiple outcome measurements, the correlation between effect sizes is neglected, potentially leading to underestimation of standard errors and false statistical significance (Tan et al., 2016). Similarly, although Howells maintained a cautious attitude toward the clinical significance of exercise intervention effects in their meta-analysis, their methodology also failed to address the challenges of multi-arm study designs. In handling multiple effect sizes within a single study, they likely adopted simple averaging or selected a single outcome, neglecting the correlation structure between effect sizes, potentially introducing bias (Howells et al., 2019).

Other studies demonstrate similar limitations. A meta-analysis on the impact of physical activity on children with ADHD/ASD found that physical activity could improve cognitive flexibility and inhibitory control, but similarly failed to adequately consider the complex data structure of multi-arm trials in the statistical analysis. Another study reported positive effects of chronic exercise on executive functions (*g* = 0.342), particularly in inhibitory control (*g* = 0.492), but its analytical method likewise ignored effect size dependencies in multi-arm designs (Liang et al., 2022).

These methodological limitations highlight the necessity of adopting three-level meta-analysis. Three-level meta-analysis can: (1) consider variation within and between studies; (2) appropriately model dependencies between effect sizes; (3) improve statistical efficiency and estimation precision; and (4) more accurately assess sources of heterogeneity. By addressing the complexity of multi-arm studies, three-level meta-analysis can provide more reliable and precise evaluations of exercise interventions in improving inhibitory control in children with ASD (Cheung, 2014, 2019).

To overcome the aforementioned limitations, this study employs a multi-level meta-analysis approach, an advanced statistical technique capable of handling dependent effect sizes, considering three-level nested structures, and simultaneously analyzing multiple outcome variables. Traditional meta-analysis assumes independence between effect sizes, but in practice, a single study often produces multiple effect sizes with dependencies between them. Multi-level meta-analysis explicitly models this dependency, allowing the inclusion of all available effect sizes, thereby maximizing the utilization of existing evidence and enhancing statistical power (Assink and Wibbelink, 2016). Additionally, multi-level meta-analysis can systematically explore potential moderating variables (such as participant characteristics, intervention parameters, study design factors), helping to identify key factors affecting intervention efficacy. Compared to traditional meta-analysis, the multi-level approach provides more precise estimates of heterogeneity sources, contributing to explaining differences between study results.

To identify the most effective exercise interventions, this study will conduct subgroup analyses comparing different exercise modalities, including mind–body exercises, structured sports (Mini Basketball, Table Tennis, Martial Arts), virtual/interactive exercises, and school-based programs. We hypothesize that structured, skill-based interventions may demonstrate superior effects on inhibitory control compared to less structured activities. These analyses will inform evidence-based exercise selection for children with ASD.

Based on the above analysis, this multi-level meta-analysis aims to comprehensively evaluate the impact of exercise interventions on inhibitory control abilities in children with ASD. First, we will synthesize existing research to assess the overall effect size of exercise interventions to determine their efficacy as an intervention approach. Second, we will explore the influence of exercise parameters on intervention effects, including frequency, intensity, duration, and total intervention period, to identify the optimal “dose” combination. Simultaneously, this study will compare the relative effects of different types of exercise, providing more precise intervention selection guidance for clinical practice. We anticipate providing a solid evidence-based foundation for developing individualized exercise intervention programs, enriching intervention strategies for inhibitory control deficits in autism, and ultimately improving the quality of life for children with ASD.

## 2 Methods

### 2.1 Registration and reporting guidelines

This systematic review and meta-analysis was prospectively registered in the International Prospective Register of Systematic Reviews (PROSPERO) with the registration number CRD420251039964. The design, implementation, and reporting of this study followed the Page et al. (2023). The PRISMA 2020 checklist was applied to ensure a rigorous and transparent approach to literature search, study selection, data extraction, risk of bias assessment, and data synthesis, aiming to enhance the reproducibility and reliability of the findings. To promote transparency and facilitate future research replication, all data analysis scripts and related materials have been openly shared on the Open Science Framework (OSF) and are available at https://osf.io/jkpcn/.

### 2.2 Search strategy

We conducted a comprehensive literature search in four electronic databases: Web of Science, PubMed, Embase, and the Cochrane Library, covering all records from inception to April 25, 2025. Both English and Chinese language studies were included to ensure broad coverage of relevant research. The search strategy was developed based on a combination of Medical Subject Headings (MeSH) (Medical subject headings - home page, 2025) and free-text terms, structured around three key concepts: (1) autism spectrum disorder (ASD), (2) exercise and sports interventions, and (3) the target population of children and adolescents. Logical operators (AND, OR) were applied to combine these components appropriately. Detailed search strings tailored to each database, including syntax, keywords, and Boolean structures, are presented in Supplementary material 1.

### 2.3 Inclusion criteria

The inclusion criteria were developed based on the PICOS framework (Amir-Behghadami and Janati, 2020). The population (P) included children or adolescents formally diagnosed with autism spectrum disorder (ASD), with no restrictions on gender, region, or language. Diagnoses could be based on DSM, ICD, or other recognized diagnostic criteria. The intervention (I) referred to any intervention primarily based on physical activity or exercise, including sports, fitness training, mind–body exercises, and virtual exercise, without restrictions on the specific type, intensity, frequency, or duration. The comparison (C) included studies with a control group, such as no intervention, usual care, or other non-exercise interventions. Multi-arm studies were also eligible if the exercise interventions in different groups were clearly distinct in type, intensity, or mode. The outcome (O) required at least one objectively measured, standardized, and quantifiable behavioral indicator of inhibitory control, such as the Stroop task, Go/No-Go task, or Flanker task. The study design (S) was limited to randomized controlled trials (RCTs) or quasi-RCTs that reported sufficient statistical data (e.g., means, standard deviations, or sample sizes) for effect size calculation.

### 2.4 Exclusion criteria

Studies were excluded if they involved mixed populations without separately reported data for the ASD subgroup. Studies involving participants outside the child or adolescent age range without subgroup data were also excluded. Studies were excluded if the exercise component could not be clearly identified or if the intervention was poorly described. Studies were excluded if the exercise intervention and the control or comparison groups did not differ meaningfully in the nature of the exercise and only varied in delivery methods, settings, or personnel. Studies were excluded if they did not report objectively measured, standardized, and quantifiable indicators of inhibitory control. Studies lacking sufficient statistical data and where the authors could not be contacted for additional information were excluded. Reviews, commentaries, case studies, conference abstracts, and non-peer-reviewed studies were also excluded.

### 2.5 Study selection and eligibility criteria

In the study selection phase, an initial screening of titles and abstracts was conducted by HYQ to exclude studies that were clearly irrelevant to the research topic. Full-text articles of the remaining records were then independently reviewed by WY and HYQ based on pre-defined inclusion and exclusion criteria to determine their eligibility. Any disagreements or uncertainties that arose during this process were resolved through discussion with SXQ until consensus was reached. All literature management and tracking were performed using Endnote 20 software. The detailed selection process is presented in the PRISMA flow diagram (Page et al., 2023).

### 2.6 Data extraction

Data extraction was independently conducted by two researchers to ensure the accuracy and consistency of the extracted information. All extracted data were subsequently reviewed and verified by another group of researchers to further ensure data quality. The extracted information included, but was not limited to, the following aspects: (1) study design and basic study characteristics; (2) participant characteristics, such as age and sex; (3) detailed descriptions of the intervention, including the type, frequency, intensity, and duration of the exercise program; (4) description and implementation details of the control group; and (5) outcome assessment methods and indicators, including measurement tools, assessment time points, and statistical data required for effect size calculations (e.g., means, standard deviations, and sample sizes). All data were organized in Excel spreadsheets for subsequent analysis. For studies that presented results in graphical formats, numerical data were extracted using tools such as WebPlotDigitizer (Drevon et al., 2017). In cases where key information was missing, the research team contacted the corresponding authors via email to request additional data. If no response was received, a reminder email was sent 48 h later. Studies for which the required data could not be obtained after these attempts were excluded from the final analysis.

### 2.7 Risk of bias evaluation

The risk of bias of the included studies was systematically assessed using the Risk of Bias 2 (ROB 2) tool recommended by the Cochrane Collaboration (Higgins et al., 2011). This tool evaluates five domains: bias arising from the randomization process, bias due to deviations from intended interventions, bias due to missing outcome data, bias in measurement of the outcome, and bias in selection of the reported result. An overall risk of bias judgment is then provided based on these domains.

### 2.8 Data transformation and effect size calculation

All meta-analyses were conducted using R software (version 4.3.0) with the metafor package. Additional analyses utilized specialized R scripts for three-level meta-analysis implementation.

Prior to conducting the meta-analysis, all raw data were subjected to rigorous standardization procedures to ensure comparability across studies. For studies reporting within-group pre-post comparisons, we extracted the pre-intervention and post-intervention means (Mpre and Mpost), standard deviations (SDpre and SDpost), and sample sizes (n1 and n2). The mean change (Mchange) and its standard deviation (SDchange) were calculated using the following formulas (Becker, 1988; Morris and DeShon, 2016) as shown in Equation (1):


(1) $$M_{\text{change}}=M_{\text{post}}-M_{\mathrm{pre}}$$

where *r* represents the correlation coefficient between pre- and post-intervention measurements. As most included studies did not report *r*, a moderate correlation assumption of *r* = 0.5 was applied in the primary analysis, following the recommendations of the Cochrane Handbook (Higgins et al., 2011). To evaluate the robustness of the findings, sensitivity analyses were conducted using alternative values of *r* = 0.6, 0.7, and 0.9 (Vasconcelos et al., 2024), examining the influence of different correlation assumptions on the overall results.

For studies comparing between-group differences, the pooled standard deviation (SDpooled) was calculated using the following formula (Equation 2): (Hedges and Olkin, 2014):


(2) $$SD_{\text{change}}=\sqrt{SD_{\mathrm{pre}}^{2}+SD_{\text{post}}^{2}-2r\cdot SD_{\mathrm{pre}}\cdot SD_{\text{post}}}$$

For studies reporting standard errors (SE) instead of standard deviations (SD), the following conversion formula was applied to ensure a consistent metric of variability across studies using Equation (3):


(3) $$\mathrm{SD}=\mathrm{SE}\times \sqrt{N}$$

Given the generally small sample sizes in the included studies, Hedges’ g was selected as the primary effect size indicator, as it provides a correction for small sample bias compared to Cohen’s d (Nelson, 2015). The formula used for calculating Hedges’ g, applicable to both within- and between-group comparisons, was as follows (Equation 4):


(4) $$\mathrm{ES}=\frac{M_{\text{post}}-M_{\mathrm{pre}}}{{\mathrm{SD}}_{\text{pooled}}}\times (1-\frac{3}{4(n_{1}+n_{2}-2)-1})$$

To facilitate interpretation and ensure consistency in effect direction, effect sizes were multiplied by −1 when necessary, so that positive values uniformly represented functional improvements or enhancements. According to the criteria proposed by Cohen (2013), effect sizes were categorized as small (ES < 0.2), medium (0.2 ≤ ES < 0.5), and large (ES ≥ 0.5).

Between-study heterogeneity was assessed using the I^2^ and Q statistics. I^2^ values of 25, 50, and 75% were interpreted as low, moderate, and high heterogeneity, respectively, and a Q-test *p*-value < 0.1 was considered indicative of significant heterogeneity. In addition, prediction intervals (PI) were calculated to provide a more comprehensive range of potential effect sizes in future studies, reflecting the overall variability, including true effect differences across studies (Nagashima et al., 2019). This information offers valuable insights for clinical decision-making and future research planning.

### 2.9 Three-level meta-analysis

In a single study, multiple effect sizes (ESs) are often reported. These effect sizes are typically statistically correlated as they originate from the same sample or measurement context. Including all these dependent effect sizes in a conventional meta-analysis may violate the assumption of independence between effect sizes across studies, potentially leading to inflated results (Harrer et al., 2021; Kadlec et al., 2023).

On the other hand, retaining only a single effect size or calculating the average of multiple effect sizes for each study might oversimplify the data structure, which could underestimate the true intervention effect by failing to capture the peak effects (Cooper et al., 2009; Van den Noortgate et al., 2013).

To address these limitations, the present study applied the three-level meta-analysis approach proposed by Assink and Wibbelink (2016), utilizing the open-source R implementation provided by Jukic et al. (2023), Xu et al. (2025). This approach accounts for the dependency among multiple effect sizes within studies (Assink and Wibbelink, 2016; Harrer et al., 2021), considers the hierarchical data structure (e.g., effect sizes nested within studies), retains multiple effect sizes from each study, improves statistical power, and provides a more realistic representation of the distribution of intervention effects (Hedges and Olkin, 2014; Assink and Wibbelink, 2016).

The three-level model explicitly distinguishes between within-study and between-study variance components. The total variance structure is decomposed into three levels (Equation 5):


(5) $$y_{\mathrm{ij}}=\mu +u_{j}+v_{\mathrm{ij}}+e_{\mathrm{ij}}$$

Where $y_{\mathrm{ij}}\;$ represents the i -th effect size in the j -th study, μ is the overall average effect, $u_{j}$ captures the between-study variance (Level 3), $\;v_{\mathrm{ij}}$ captures the within-study variance (Level 2), and $e_{\mathrm{ij}}$ represents the sampling error (Level 1).

The corresponding variance structure is defined as (Equation 6):


(6) $$\mathrm{Var}(y_{\mathrm{ij}})=s_{\mathrm{ij}}^{2}+\sigma^{2}+\tau^{2}$$

Where $s_{\mathrm{ij}}^{2}$ is the known sampling variance, σ2 represents the within-study variance, and τ2 represents the between-study variance (Cheung, 2014, 2019).

The present analysis was conducted following the framework proposed by Assink and Wibbelink (2016) and implemented using the R scripts provided by Jukic et al. (2023). Model parameters were estimated using restricted maximum likelihood (REML), and model robustness was cross-validated using maximum likelihood (ML). Statistical significance and confidence intervals (CIs) were calculated based on the t-distribution (Jukic et al., 2023).

All three-level meta-analyses were performed using the metafor package in R (version 4.3.0, R Core Team) (Viechtbauer, 2010). This approach allowed the inclusion of multiple reported effect sizes from each study, offering a more comprehensive assessment of the true distribution of intervention effects and providing stronger evidence for result interpretation and intervention optimization.

By nesting multiple measurements and comparisons within each study, the observed variance was decomposed into three levels: sampling variance (Level 1), within-study variance (Level 2), and between-study variance (Level 3). This hierarchical variance decomposition allowed for controlling the dependency among effect sizes within studies (Cheung, 2014, 2019).

### 2.10 Subgroup analysis and meta-regression

To explore potential sources of heterogeneity and investigate the effects of study-level moderators, both subgroup analyses and meta-regression were conducted. Multiple modeling approaches were compared, including simple linear meta-regression, polynomial meta-regression, and restricted cubic spline (RCS) regression (Harrell, 2015). The selection of the final model was based on a combination of goodness-of-fit criteria and substantive interpretability (Nuzzo et al., 2024).

Given that linear models may impose unrealistic assumptions of indefinitely increasing or decreasing trends, particularly in the context of exercise interventions, this study prioritized the interpretation of nonlinear models, such as polynomial and spline regressions. Nevertheless, results from linear meta-regression models were also reported for comparative purposes.

Meta-regression allows for the prediction and evaluation of how study-level variables may influence the magnitude of effect sizes across different conditions or subgroups. All meta-regression analyses were performed using the metafor package in R, and the resulting models were visualized using the ggplot2 package (Wickham, 2011), ensuring clear and interpretable graphical representations of the modeled relationships.

### 2.11 Publication bias and sensitivity analysis

To evaluate potential publication bias, this study employed contour-enhanced funnel plots (Peters et al., 2008) in combination with Egger’s regression tests (Egger et al., 1997). A *p*-value greater than 0.05 in Egger’s test was interpreted as no significant indication of publication bias. These assessments were carried out separately at both the within-study level (Level 2) and the between-study level (Level 3). Funnel plots and Egger’s regression were used to visually and statistically assess the symmetry of the effect size distribution, providing evidence regarding the presence or absence of publication bias among the included studies (Afonso et al., 2024).

Considering that this study applied a three-level meta-analytic model, which accounts for multiple effect sizes nested within individual studies, publication bias analyses were conducted at both levels. Specifically, the within-study analysis (Level 2) considered all reported effect sizes, while the between-study analysis (Level 3) was based on the mean effect size calculated for each study.

In addition to publication bias assessment, this study also performed influence diagnostics to identify potential outliers and influential cases. Hat values (Viechtbauer and Cheung, 2010), Cook’s distances (Viechtbauer and Cheung, 2010), and studentized residuals (Robinson et al., 1984) were computed to detect high-leverage points, outliers, and influential observations at both Level 2 and Level 3. Furthermore, leave-one-study-out sensitivity analyses were conducted at both levels to examine the robustness of the overall results by assessing how the exclusion of each study individually influenced the pooled estimates (Harrer et al., 2021).

### 2.12 Certainty assessment

To enhance the transparency and credibility of result interpretation, this study incorporated both the quality appraisal of included studies and the overall assessment of evidence certainty. The GRADE (Grading of Recommendations Assessment, Development and Evaluation) framework (Guyatt et al., 2011; Schünemann et al., 2019; Piggott et al., 2020) was applied to evaluate the certainty of evidence. Widely used in systematic reviews and guideline development, GRADE classifies evidence certainty into five levels: high, moderate, low, very low, and extremely low.

Following the GRADE criteria, the certainty of evidence was systematically judged based on five key domains: risk of bias, inconsistency of results, indirectness of evidence, imprecision of effect estimates, and risk of publication bias. This structured approach provided a comprehensive evaluation of the overall strength and reliability of the findings.

## 3 Result

### 3.1 Search results

**Figure 1:**
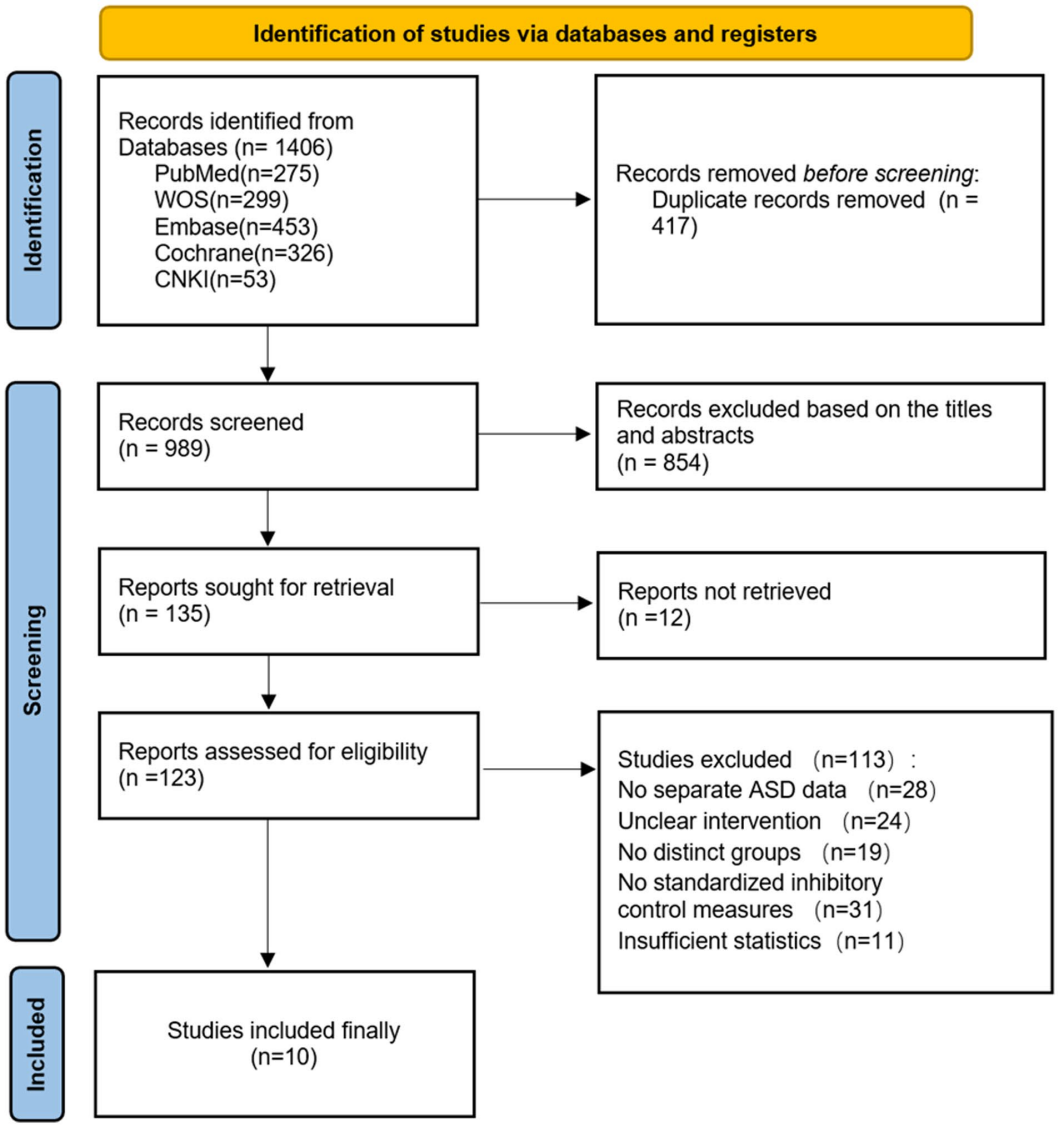
Flowchart.

Following the PRISMA statement guidelines, we conducted literature screening and selection. Through systematic searches of PubMed (*n* = 275), Web of Science (*n* = 299), Embase (*n* = 453), Cochrane Library (*n* = 326), and CNKI (*n* = 53) databases, a total of 1,406 initial articles were retrieved. After removing 417 duplicate records, we conducted preliminary screening of the titles and abstracts of 989 articles, excluding 854 articles that did not meet the inclusion criteria. Subsequently, we attempted to obtain full texts of the remaining 135 articles, of which 12 could not be accessed. Detailed assessment was conducted on the 123 successfully obtained full texts, resulting in the exclusion of 113 articles that did not meet the criteria. The main reasons for exclusion included: no separate ASD population data (*n* = 28), unclear intervention description (*n* = 24), no significant distinction between control and intervention groups (*n* = 19), no standardized inhibitory control measurements (*n* = 31), and insufficient statistical data (*n* = 11). Ultimately, 10 studies meeting all inclusion criteria were included in this Meta-analysis. The included studies were all randomized or quasi-randomized controlled trials evaluating the effects of exercise interventions on inhibitory control abilities in children and adolescents with ASD. The entire screening process was completed by two independent researchers, with disagreements resolved by a third researcher. Figure 1 illustrates the detailed literature screening process. The table of literature characteristics is presented in Appendix I.

### 3.2 Study characteristics

This study ultimately included 10 empirical studies published between 2013 and 2023 that met the criteria, covering four countries: China (*n* = 7), South Korea (*n* = 1), the United States (*n* = 1), and Iran (*n* = 1). Participants in these studies were all children or adolescents formally diagnosed with ASD, with mean ages ranging from 4.79 to 14.3 years, specifically encompassing early childhood (3–6 years), childhood (6–12 years), and early adolescence (8–18 years). The proportion of females ranged from 0 to 24%, demonstrating a predominantly male gender distribution, which is consistent with the general gender distribution in the ASD population.

Sample sizes in the included studies varied slightly between experimental and control groups, with experimental group sample sizes ranging from 11 to 23 participants and control group sample sizes ranging from 11 to 22 participants. Intervention types were diverse, including traditional mind–body exercises (such as Nei Yang Gong), structured physical activities [such as Mini Basketball (MB), Table Tennis (TT), Martial Arts (MA)], technology-assisted virtual exercises (such as interactive games, virtual cycling), and school-based physical activity programs (such as the SPARK curriculum). Control group designs were equally diverse, including treatment as usual (such as behavioral or educational interventions), other types of physical activities (such as regular physical exercise), and non-exercise activities (such as walking).

Intervention protocols exhibited certain differences in duration, frequency, and intensity. Intervention periods ranged from 2 to 13 weeks, with intervention frequencies of 2 to 5 sessions per week, each lasting 30 to 70 min. These differences reflect the diversity in exercise intervention design across different studies, aiming to promote cognitive and behavioral improvements in participants through various forms of exercise.

All included studies employed at least one standardized, objective measure of inhibitory control as an outcome assessment tool. These assessment tools included the Stroop task, Go/No-Go task (GNG), Flanker task, and other validated behavioral assessment tools such as the Children’s Color Trails Test (CCTT-T2), Childhood Executive Functioning Inventory (CHEXI), Wisconsin Card Sorting Test (WCST), Working Card Task (WCTS), Hearts and Flowers task, Behavior Rating Inventory of Executive Function (BRIEF-2), Tower of London task (TOL), and Stroop Color-Word Test (SCWT). All these tools provide quantifiable behavioral data, ensuring consistency with the predetermined inclusion criteria for this.

### 3.3 Study quality and risk of bias

**Figure 2:**
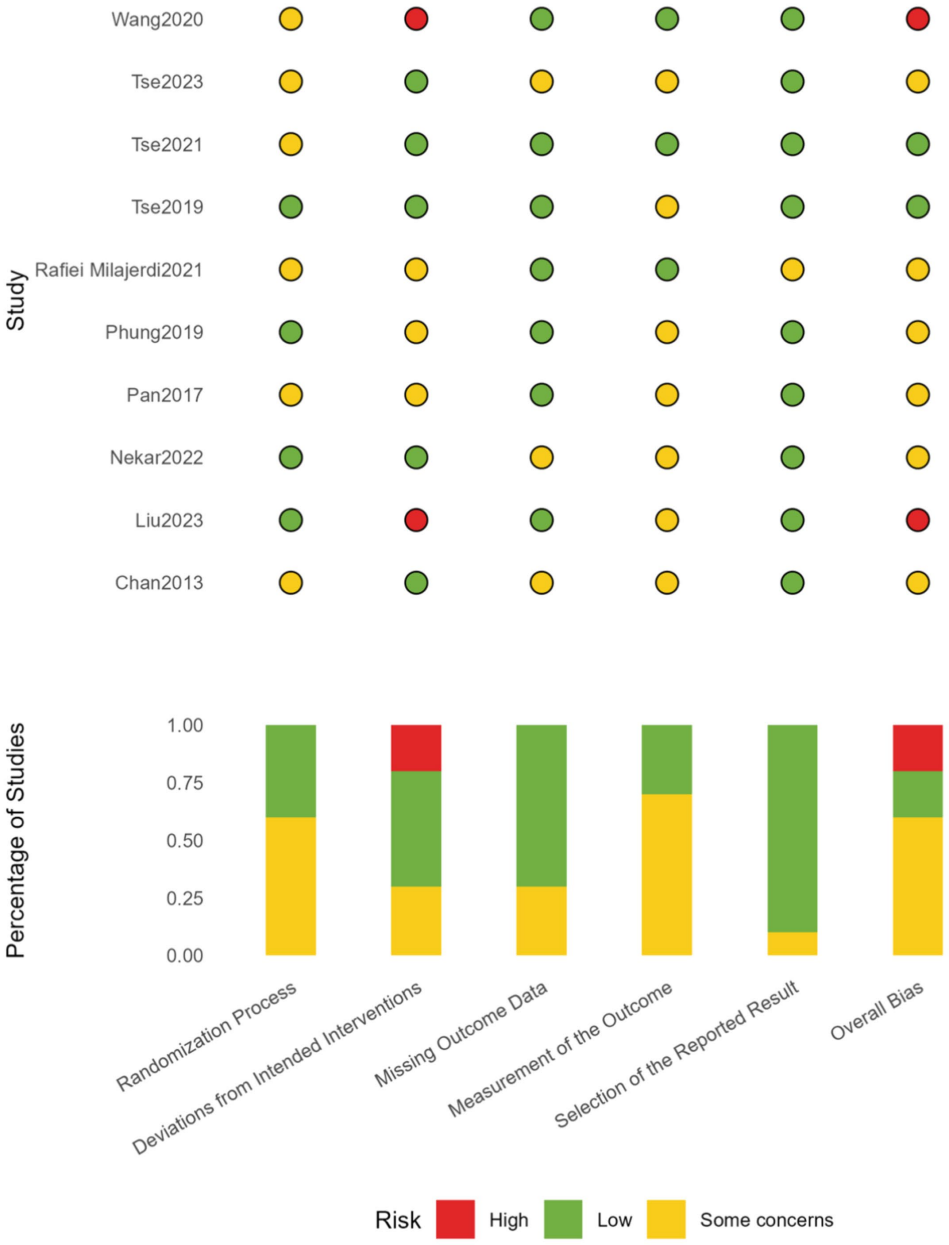
ROB2.

Using the ROB2 tool, we conducted a risk of bias assessment for the 10 included exercise intervention studies. Regarding the randomization process, 4 studies were classified as low risk, while 6 demonstrated some concerns; for deviations from intended interventions, 4 studies were assessed as low risk, 3 showed some concerns, and 3 were high risk, with Wang et al. (2022) and Liu et al. (2023) rated as high risk due to intervention adherence issues; for missing outcome data, 7 studies were low risk and 3 presented some concerns; in the outcome measurement dimension, 5 studies were low risk and 5 showed some concerns, primarily affected by difficulties in implementing blinding procedures; selection of reported results performed best, with 9 studies categorized as low risk and only 1 showing some concerns; in the overall assessment, 4 studies were deemed low risk, 4 showed some concerns, and 2 were classified as high risk. Overall, exercise intervention studies primarily face challenges in intervention adherence and implementation of blinding procedures, reflecting inherent methodological limitations in this type of research. However, most studies adhered to good practice standards regarding randomization and outcome reporting (see Figure 2).

**Figure 3:**
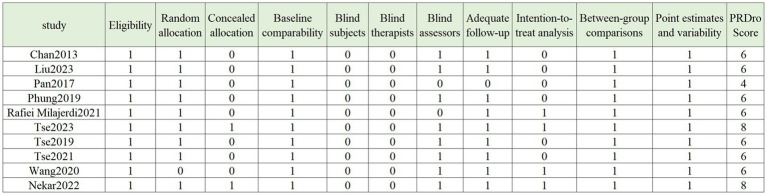
PEDro scale.

The PEDro scale (Physiotherapy Evidence Database scale) was used to assess the quality of the studies included in this research. The PEDro scale is a widely used tool for evaluating the quality of randomized controlled trials (RCTs), covering 11 key items such as study design, allocation methods, blinding, follow-up, and reporting of outcomes. Each item is scored as “Yes” or “No,” and the final score reflects the overall quality of the study. The total score ranges from 0 to 10, with higher scores indicating stronger internal validity and more reliable evidence. In this study, PEDro scores ranged from 4/10 to 9/10, with the results showing that most studies had high quality in experimental design and data analysis, though some limitations were noted, such as incomplete blinding and insufficient follow-up (Figure 3).

### 3.4 Main effects and subgroup analysis

**Figure 4:**
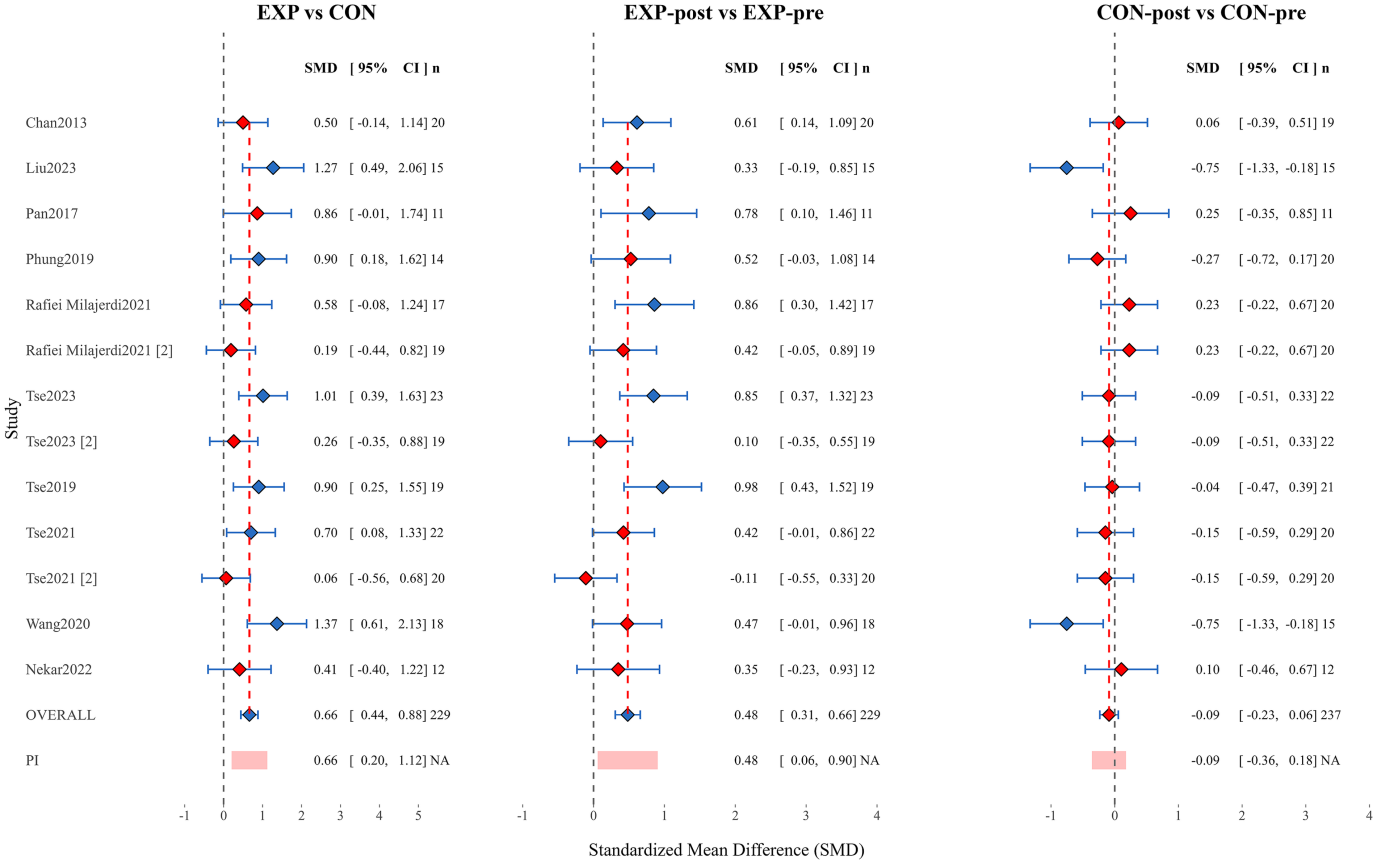
Forest plot.

As shown in the Figure 4, this meta-analysis forest plot presents three core comparisons: between experimental and control groups (EXP vs. CON), pre-post comparison within the experimental group (EXP-post vs. EXP-pre), and pre-post comparison within the control group (CON-post vs. CON-pre).

The pooled standardized mean difference (SMD) for the experimental versus control group comparison (EXP vs. CON) was 0.66 (95% CI [0.44, 0.88]; *n* = 229), indicating a moderate positive effect of experimental interventions compared to control conditions, with statistical significance. Among individual studies, Liu et al. (2023) reported larger effect sizes (SMDs of 1.37 and 1.27 respectively), while Tse et al. (2021) and Rafiei Milajerdi et al. (2021) showed smaller effects (SMDs of 0.06 and 0.19 respectively). The prediction interval (PI) was 0.66 (95% CI [0.20, 1.12]), further confirming the robustness of the effect.

Analysis of the experimental group pre-post comparison (EXP-post vs. EXP-pre) revealed a pooled SMD of 0.48 (95% CI [0.31, 0.66]; *n* = 229), confirming that experimental interventions led to a small-to-moderate significant improvement in participants’ condition. Among these, studies by Tse et al. (2019) and Rafiei Milajerdi et al. (2021) demonstrated the most significant pre-post changes (SMDs of 0.98 and 0.86 respectively), while only the study by Tse et al. (2021) showed a slight negative effect (SMD = −0.11; 95% CI [−0.55, 0.33]), which was not statistically significant. The prediction interval result of 0.48 (95% CI [0.06, 0.90]) indicates that in 95% of contexts, interventions may produce positive effects ranging from small to moderate magnitude.

Meta-analysis results for the control group pre-post comparison (CON-post vs. CON-pre) showed a pooled SMD of −0.09 (95% CI [−0.23, 0.06]; *n* = 237), with the confidence interval including zero, indicating no significant change in participants’ overall condition under control conditions. Notably, control groups in studies by Liu et al. (2023) and Wang et al. (2022) exhibited larger negative changes (both with SMDs of −0.75), while control groups in studies by Pan et al. (2017) and Rafiei Milajerdi et al. (2021) showed slight improvements (SMDs of 0.25 and 0.23 respectively), reflecting between-study heterogeneity in control group condition changes.

Overall, the forest plot analysis results strongly support the efficacy of experimental interventions. The experimental group demonstrated a moderate significant effect compared to the control group (SMD = 0.66), with notable improvement within the experimental group from pre to post-intervention (SMD = 0.48), while the control group showed no significant change during the corresponding period (SMD = −0.09). This pattern of results excludes the influence of time effects or other non-specific factors, confirming that the observed effects can be attributed to the experimental intervention itself. The heterogeneity in effect sizes between studies suggests that intervention effects may be moderated by factors such as study population characteristics, intervention protocols, and measurement tools, which should be fully considered in clinical applications.

**Figure 5:**
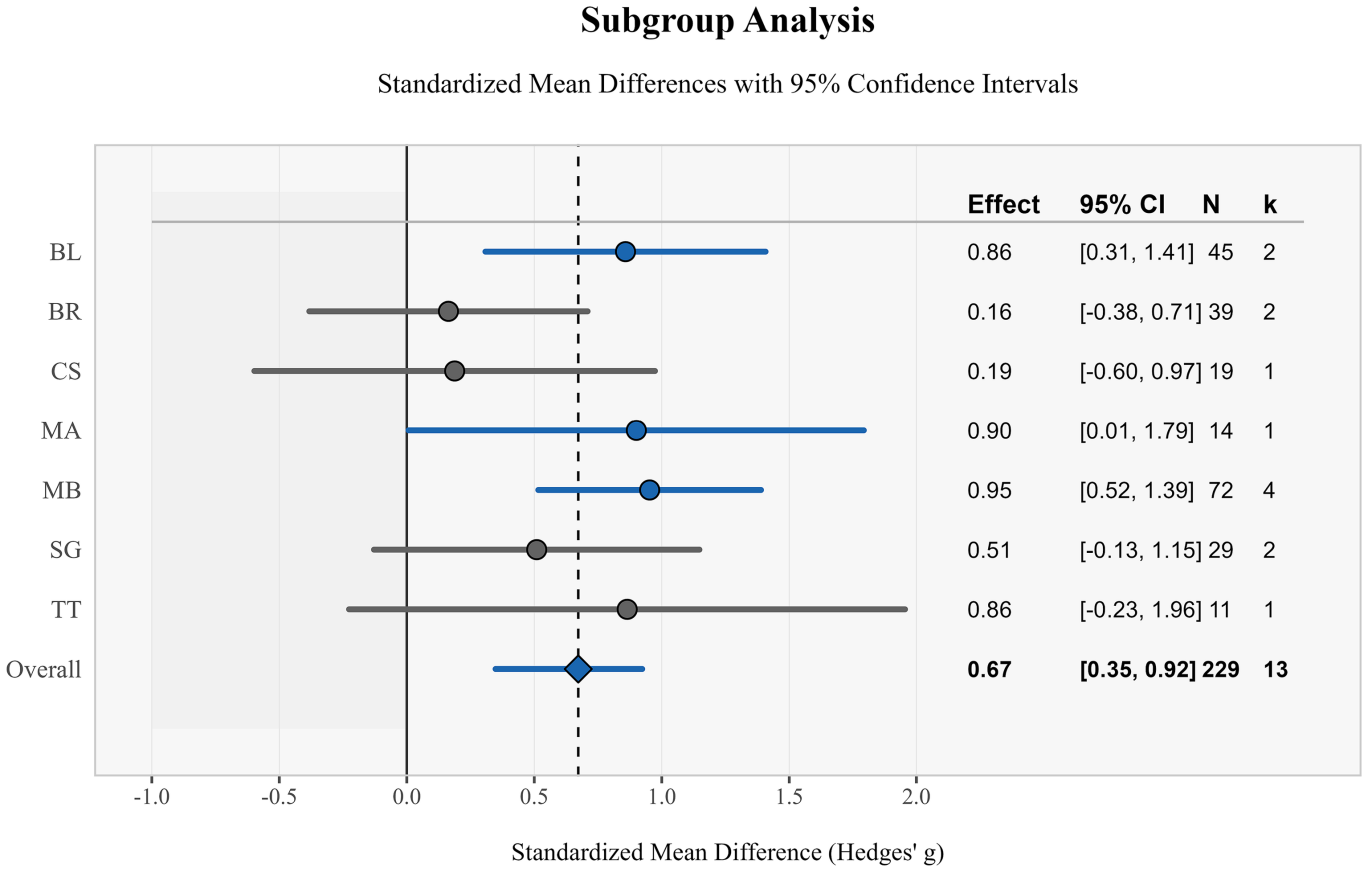
Subgroup analysis of forest maps.

This study evaluated the effects of different types of exercise interventions on children through subgroup analysis. Results showed varying degrees of effect sizes in standardized mean differences (Hedges’ g) across exercise interventions, with an overall effect size of 0.67 (95% CI: [0.35, 0.99]), indicating a moderate positive effect of exercise interventions overall (Figure 5).

MB demonstrated the strongest effect, with a standardized mean difference of 0.95 (95% CI: [0.52, 1.39]), based on 4 studies (*n* = 72), with confidence intervals not containing zero, indicating statistical significance of the effect. This was followed by MA, with an effect size of 0.90 (95% CI: [0.01, 1.79]), which remained significant despite the small sample size (*n* = 14). BL and TT exhibited similar effect sizes of 0.86 (95% CI: [0.31, 1.41]) and 0.86 (95% CI: [−0.23, 1.96]) respectively, but notably, the confidence interval for TT crossed zero, indicating its effect was not statistically significant.

SG demonstrated a moderate effect size of 0.51 (95% CI: [−0.13, 1.15]), but similarly did not reach statistical significance. CS and BR showed the smallest effects, at 0.19 (95% CI: [−0.60, 0.97]) and 0.16 (95% CI: [−0.38, 0.71]) respectively, with both confidence intervals containing zero, suggesting the effects of these interventions may not be significant.

**Figure 6:**
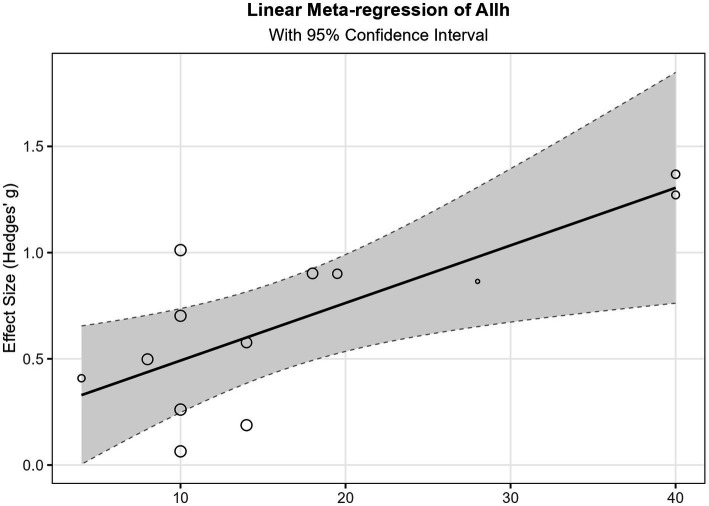
The total intervention duration of linear regression.

Overall, these subgroup analysis results suggest that structured exercise interventions such as MB, MA, and BL may provide more significant benefits than other forms of exercise. MB in particular, with its larger sample size and narrow confidence interval, provided the strongest evidence in support. Notably, although some intervention methods did not reach statistical significance, point estimates for all interventions were positive, indicating potential positive trends. These findings provide important references for the design and implementation of exercise intervention programs for children (Figure 6).

### 3.5 Linear and non-linear regression

**Figure 7:**
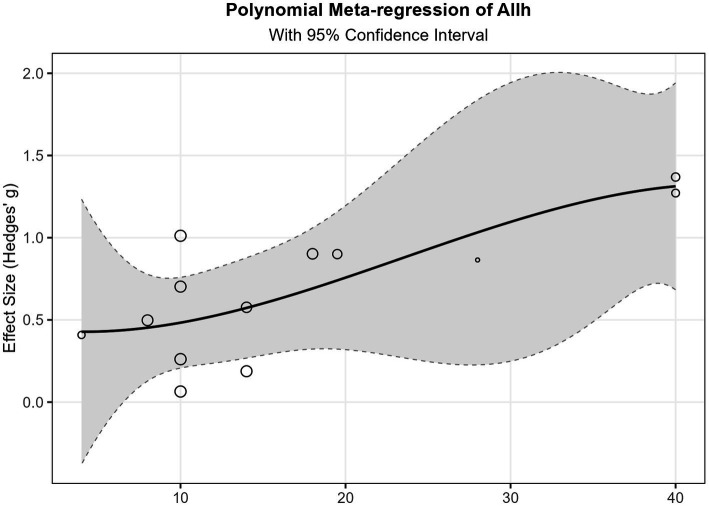
Spline regression of total intervention duration.

**Figure 8:**
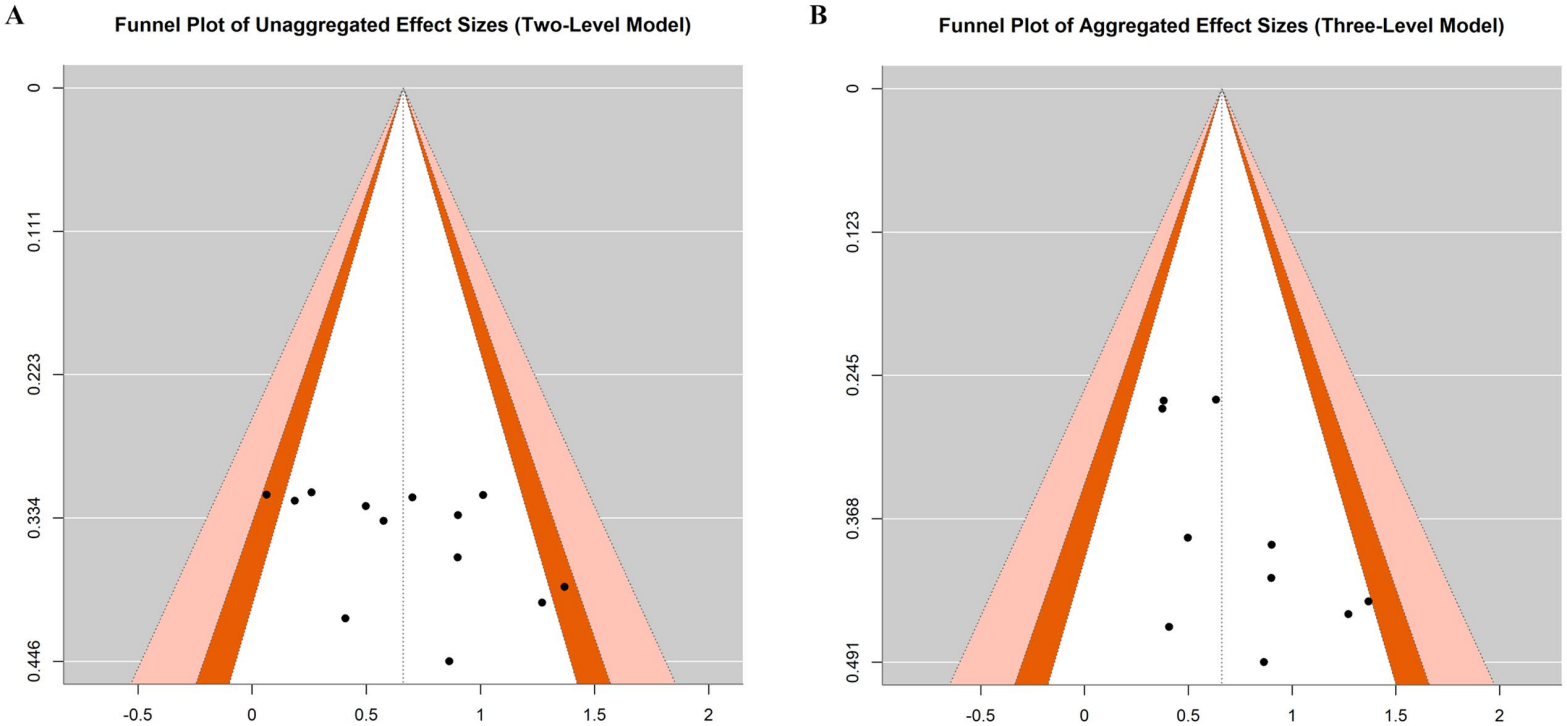
Funnel diagram.

This study explored the relationships between five predictor variables (age, training weeks, training sessions, training minutes, and total training duration) and effect sizes using three methods: linear meta-regression, polynomial meta-regression, and restricted cubic spline meta-regression. Refer to Figures 7, 8 and Appendix 1 for details.

Total training duration showed statistical significance in the linear model (*β* = 0.027, *p* = 0.015), indicating that effect sizes exhibited a significant increasing trend with increased total training duration. Specifically, for each additional hour of total training duration, the effect size increased by an average of 0.027 units. Additionally, the overall restricted cubic spline regression model for total training duration also reached significance (*p* = 0.047), further supporting the important influence of this variable on outcomes, although the specific form of its non-linear relationship remains unclear. The age variable demonstrated marginal significance in the linear model (*β* = −0.091, *p* = 0.083), with a negative coefficient suggesting that effects may decrease with increasing age. In the restricted cubic spline regression, the intercept term for age reached significance (*p* = 0.014), and its linear term approached significance (*p* = 0.091). These results consistently indicate that age is an important factor influencing effect sizes. Training weeks approached significance in the linear model (*β* = 0.050, *p* = 0.051), with a positive coefficient suggesting that extending training duration may help improve effectiveness. However, its non-linear relationship model did not show significant results, and fitting failed in the restricted cubic spline model, possibly due to data distribution characteristics or sample size limitations.

Training minutes reached marginal significance for all terms in polynomial regression analysis (*p* values between 0.07–0.09), suggesting a potentially complex non-linear relationship. Specifically, the coefficients of its cubic polynomial model indicate that effect sizes may initially increase with training minutes, then decrease after reaching a critical point, and finally slightly increase again in the high-value region. This complex non-linear relationship suggests the existence of an optimal training duration interval. Training sessions showed no significant findings in any analyzable models and could not be analyzed in some models due to insufficient unique values. This indicates that simply increasing training frequency may not be a key factor in improving effectiveness.

Residual heterogeneity tests for all models were non-significant (*p* > 0.05), indicating appropriate model fitting and that between-study heterogeneity was well explained. Variance components τ^2^ were small for most variables, particularly for total training duration (τ^2^ = 0), further confirming the explanatory power of this variable. However, the relatively small sample size included in the meta-regression analysis (*k* = 13) may have affected the statistical power and robustness of results for some complex models (such as polynomial regression and restricted cubic spline regression), especially for findings that only reached marginal significance. This may also explain why some variables showed inconsistent result patterns across different models. See Appendix 1 for details.

Synthesizing the above results, this study indicates that: total training duration is the most robust predictor of effect sizes, showing a significant positive linear relationship; age may have a negative impact, with effects weakening as age increases; training weeks have a potential positive contribution; while training minutes may have an optimal interval. These findings provide important references for intervention program design, particularly in planning total intervention duration and selecting target age groups.

### 3.6 Publication bias and evidence grading

To evaluate potential publication bias, we conducted regression tests for funnel plot asymmetry and visual inspections of funnel plots based on both unaggregated (two-level) and aggregated (three-level) models. In the two-level model, the regression test indicated no statistically significant asymmetry (*z* = 1.803, *p* = 0.071), although the funnel plot showed a relatively balanced distribution of effect sizes (Figure 8). Similarly, in the three-level aggregated model, the regression test also did not reach statistical significance (*z* = 1.868, *p* = 0.062), suggesting no strong evidence of publication bias (Figure 8).

Despite the marginal *p*-values in both models, the visual inspection of the funnel plots did not reveal substantial asymmetry. These results suggest that the likelihood of publication bias affecting the overall findings is relatively low.

A systematic evaluation of the evidence quality was conducted using the GRADE framework. Randomized controlled trials received an initial “high” rating, while quasi-randomized studies started at “low” certainty, following standard GRADE methodology. GRADE assessments were conducted separately for each outcome measure of inhibitory control. Despite these initial ratings, the final certainty of evidence was downgraded to “low” for all outcomes due to multiple downgrading factors. The primary reasons for downgrading included: (1) risk of bias, with two studies (Wang and Liu) rated as high risk, primarily concerning intervention adherence and implementation of blinding procedures; (2) inconsistency of results, with notable variations in effect sizes across studies (SMDs ranging from 0.06 to 1.37) and inconsistent outcomes in control groups; (3) imprecision of effect estimates, with most studies having small sample sizes (11–23 participants per group) and several subgroup interventions (CS, BR, SG, TT) showing confidence intervals crossing zero. Although evidence of a dose–response relationship was provided by the significant positive correlation between total training duration and effect size (*β* = 0.027, *p* = 0.015), this only partially offset the impact of downgrading factors. Across all outcome measures, the low certainty evidence indicates limited confidence in the effect estimates for exercise interventions improving inhibitory control abilities in children with ASD, suggesting that the true effect may substantially differ from the estimated effect. Future high-quality, large-sample studies are crucial for enhancing the certainty of evidence in this field. See Appendix for outcome-specific GRADE assessment tables.

Comprehensive sensitivity analyses were conducted to assess the robustness of our findings. Influence diagnostics including hat values, Cook’s distances, and studentized residuals were computed at both Level 2 and Level 3 to identify potential outliers and influential cases. Leave-one-study-out sensitivity analyses were performed to examine how the exclusion of individual studies affected the pooled estimates. The sensitivity analyses revealed no studies with extremely high influence metrics that would warrant exclusion, and the overall effect size remained stable across all iterations, confirming the robustness of our meta-analytic findings.

## 4 Discussion

### 4.1 Research objectives and main findings summary

This study aimed to systematically evaluate the effects of exercise interventions on inhibitory control abilities in children and adolescents with ASD. Through meta-analysis of 10 randomized or quasi-randomized controlled trials, this study found that exercise interventions have a large effect size positive impact on inhibitory control abilities in children and adolescents with ASD (SMD = 0.66, 95% CI [0.44, 0.88]). According to effect size classification standards (small effect: ES < 0.2; medium effect: 0.2 ≤ ES < 0.5; large effect: ES ≥ 0.5), the 0.66 standardized mean difference exhibited by the experimental group compared to the control group reflects the significant benefits of exercise interventions. Further analysis indicated that the experimental group demonstrated a medium effect size improvement from pre- to post-intervention (SMD = 0.48, 95% CI [0.31, 0.66]), while the control group showed no significant change during the corresponding period (SMD = −0.09, 95% CI [−0.23, 0.06]), with an effect size less than 0.2. This pattern excludes the influence of time effects or other non-specific factors, confirming that the observed effects can be attributed to the exercise intervention itself.

Subgroup analysis further revealed differential effects of various exercise intervention types, with MB (SMD = 0.95), MA (SMD = 0.90), and BL (SMD = 0.86) all demonstrating large effect size positive impacts. Meta-regression analysis confirmed that total training duration (*β* = 0.027, *p* = 0.015) is the most robust predictor of effect size, and age may have a negative influence (*β* = −0.091, *p* = 0.083), suggesting that the effect may decrease with increasing age.

### 4.2 Effect differences among various exercise intervention types

This study found significant differences in the effects of different exercise intervention types on inhibitory control abilities in children with ASD. MB, as the intervention type with the strongest effect in the study (SMD = 0.95), may benefit from its comprehensive nature—it not only requires participants to engage in physical activity but also demands sustained attention, rapid decision-making, and motor inhibition. When children with ASD face dynamically changing court situations, they need to inhibit inappropriate responses (such as not passing or shooting at appropriate times) while executing appropriate behaviors. This “instant decision-action” training mode may directly strengthen inhibitory control neural circuits. Similarly, the significant effect of MA interventions (SMD = 0.90) may derive from their unique training characteristics, including highly structured movement sequences, strict disciplinary requirements, and clear start-stop commands. These characteristics particularly align with the cognitive needs of children with ASD, providing predictability while also training motor inhibition abilities. Traditional martial arts emphasize the training concept of “mind controlling movement”; this practice method that combines internal and external elements may complement the rapid responses in basketball, jointly promoting the enhancement of inhibitory control abilities. BL (SMD = 0.86) also demonstrated a large effect size, possibly because the riding process requires continuous balance, direction control, and speed regulation; this multi-task coordination training poses ongoing challenges to the inhibitory control functions of the prefrontal cortex. In contrast, CS (SMD = 0.19) and BR (SMD = 0.16) exhibited weaker effects, reaching only small effect size levels, which may indicate that simple repetitive activities or exercises lacking clear cognitive challenges have limited promoting effects on inhibitory control. This finding suggests that exercise interventions designed for the ASD population should transcend mere physical activity and need to integrate cognitive challenge elements, especially tasks that can train response inhibition, selective attention, and conflict resolution.

### 4.3 Moderating effects of intervention parameters on outcomes

Meta-regression analysis results revealed significant moderating effects of intervention parameters on outcomes. Total training duration, as the most robust predictor (*β* = 0.027, *p* = 0.015), indicates a “dose–response” relationship of intervention time—for each additional hour of total training duration, the effect size increases by an average of 0.027 units. This finding aligns with cognitive neuroplasticity theory, namely that sustained, sufficient training is a key condition for inducing functional reorganization of the nervous system. Notably, training minutes displayed complex non-linear relationships in polynomial regression, suggesting the existence of an optimal training duration interval. This phenomenon may reflect the attention characteristics of children with ASD—training that is too short may struggle to produce sufficient neural stimulation, while training that is too long may lead to attention fatigue or overstimulation, reducing training effectiveness. The negative influence of the age factor (*β* = −0.091, *p* = 0.083) suggests that intervention effects may weaken with increasing age. This phenomenon may be related to sensitive periods of neural development—younger children with ASD may be in a critical period of inhibitory control development, with higher neuroplasticity, thus showing stronger responsiveness to exercise interventions. However, it should be noted that the sample size included in this study’s meta-regression analysis was relatively small (*k* = 13), which may have affected the robustness of statistical results, especially for findings that only reached marginal significance levels. Therefore, the interpretation of these moderating effects should be cautious, and future research requires validation with larger samples.

### 4.4 Physiological mechanisms of exercise improving inhibitory control

The biological mechanisms behind this study’s results may involve multi-level neurophysiological changes that interrelate to form an integrated mechanism network for exercise interventions improving inhibitory control. At the molecular level, exercise interventions may enhance neuronal synaptic plasticity by promoting the activation of brain-derived neurotrophic factor (BDNF) and its receptor tyrosine kinase B (TrkB) signaling pathway, especially in brain regions closely related to inhibitory control such as the prefrontal cortex and basal ganglia (Huang et al., 2014). These molecular-level changes in turn affect neural circuit function, manifested as enhanced functional connectivity of prefrontal-striatal circuits observed in functional neuroimaging studies (Di Martino et al., 2011). Specifically, exercise interventions may optimize neural network functions related to inhibitory control by regulating information transmission between the dorsolateral prefrontal cortex (DLPFC), anterior cingulate cortex (ACC), and basal ganglia, and this network is precisely the neural basis of the behavioral improvements observed in this study (Becker et al., 2016). At a deeper level, these functional changes are closely related to neurotransmitter level regulation; exercise interventions may improve GABA (*γ*-aminobutyric acid) neurotransmission dysfunction commonly found in children with ASD (Lyssikatos et al., 2015). As the main inhibitory neurotransmitter, GABA is crucial for maintaining excitation-inhibition balance in the brain, and this balance is the physiological basis for effective inhibitory control. In addition to these central nervous system changes, exercise interventions may also create a more favorable physiological environment for inhibitory control by regulating autonomic nervous system function, particularly enhancing parasympathetic activity and reducing the hypersensitivity and sensory hyperreactivity common in children with ASD (Lyssikatos et al., 2015). This multi-level mechanism interaction from the autonomic nervous system to the central nervous system, from the molecular level to network function, may collectively constitute the comprehensive biological basis for exercise interventions improving inhibitory control abilities in children with ASD.

### 4.5 Clinical application value and implementation recommendations

Based on the findings of this study, we propose the following targeted and practical clinical application recommendations. Regarding exercise intervention type selection, priority should be given to highly structured exercise types that combine cognitive challenges with physical activity, such as MB (SMD = 0.95) and MA (SMD = 0.90). These exercise forms not only provide physical exercise but also simultaneously train core components of inhibitory control such as attention switching, response inhibition, and conflict resolution. In terms of intervention parameter optimization, our meta-regression results demonstrate that total training duration is the strongest predictor of effect size (*β* = 0.027, *p* = 0.015), indicating that each additional hour of total training increases effect size by an average of 0.027 units. The polynomial regression analysis of training minutes revealed a complex non-linear relationship (*p*-values 0.07–0.09), suggesting an optimal training session duration interval that balances training effects and attention maintenance, with effectiveness initially increasing, then decreasing after reaching a critical point, and slightly increasing again at higher values. Considering the moderating role of age, with our analysis showing a negative coefficient (*β* = −0.091, *p* = 0.083) indicating effect weakening with increasing age, it is recommended to adopt differentiated intervention strategies that emphasize early intervention for younger individuals while increasing training intensity and cognitive challenge difficulty for older individuals to overcome age-related effect reduction. Additionally, the near-significant positive effect of training weeks (*β* = 0.050, *p* = 0.051) supports extending intervention duration, while the lack of significance for training frequency suggests that simply increasing session frequency may not be the key factor for improving effectiveness.

### 4.6 Research limitations and future research directions

Although this study provides valuable findings, several important limitations should be carefully considered when interpreting the results. The sample size of included studies is relatively limited and geographically imbalanced, which may restrict the generalizability of the results. The methodological quality of included studies varies, with some studies showing high risk of bias in randomization processes, intervention adherence, and blinding implementation, which may affect the reliability of the combined effect estimates. The measurement tools for inhibitory control are inconsistent across studies, covering various methods from behavioral tasks to questionnaire assessments; this heterogeneity may mask differential effects of specific interventions on specific inhibitory control components. Based on these limitations, future research should focus on the following directions: Conducting more high-quality, large-sample, multi-center randomized controlled trials, especially in non-Asian countries and regions, to enhance the generalizability of evidence; employing multimodal assessment methods, combining behavioral measurements, neurophysiological indicators, and ecological assessments to comprehensively capture changes in different dimensions of inhibitory control; exploring differential responses of different ASD subtypes to different exercise interventions to provide a basis for precision intervention; extending follow-up periods to evaluate the long-term effects of exercise interventions; and exploring synergistic effects between exercise interventions and other intervention methods to develop more comprehensive integrated intervention plans.

## 5 Conclusion

This meta-analysis provides strong evidence supporting exercise interventions for improving inhibitory control abilities in children and adolescents with ASD. Results indicate that structured exercise interventions (particularly MB, MA, and BL) have large effect size positive impacts, and total intervention duration and participant age are important moderating factors affecting outcomes. These findings provide important implications for clinical practice, emphasizing the value of integrating exercise interventions into comprehensive intervention systems for ASD, especially for improving the inhibitory control component of executive function. Despite certain limitations, the results of this study still provide scientific support for the effectiveness of exercise interventions as a strategy for promoting cognitive function in children with ASD and offer clear directions for future research.

## Data Availability

The raw data supporting the conclusions of this article will be made available by the authors, without undue reservation.
