# Supplementary material for: Multilevel meta-analysis of the effect of exercise intervention on inhibitory control in children with ASD
Source: Front Psychol. 2025 Oct 9;16:1632555. doi: 10.3389/fpsyg.2025.1632555 (PMC12546240; doi:10.3389/fpsyg.2025.1632555)
Supplement: Supplementary file 1 [file Table_1.DOCX]

## Search formulation

Pubmed（275）

#1： ((((Autism Spectrum Disorder[MeSH Terms]) OR (Autism Spectrum Disorders)) OR (Autistic Spectrum Disorder)) OR (Autistic Spectrum Disorders)) OR (Disorder, Autistic Spectrum)

#2 ：(((((((((((Exercise Therapy[MeSH Terms]) OR (Remedial Exercise)) OR (Exercise, Remedial)) OR (Exercises, Remedial)) OR (Remedial Exercises)) OR (Therapy, Exercise)) OR (Exercise Therapies)) OR (Therapies, Exercise)) OR (Rehabilitation Exercise)) OR (Exercise, Rehabilitation)) OR (Exercises, Rehabilitation)) OR (Rehabilitation Exercises)

#3 ：((((Sports[MeSH Terms]) OR (((Sport[Title/Abstract]) OR (Athletics[Title/Abstract])) OR (Athletic[Title/Abstract]))) OR ((Exercise Therapy[MeSH Terms]) OR (((((((((((Remedial Exercise[Title/Abstract]) OR (Exercise, Remedial[Title/Abstract])) OR (Exercises, Remedial[Title/Abstract])) OR (Remedial Exercises[Title/Abstract])) OR (Therapy, Exercise[Title/Abstract])) OR (Exercise Therapies[Title/Abstract])) OR (Therapies, Exercise[Title/Abstract])) OR (Rehabilitation Exercise[Title/Abstract])) OR (Exercise, Rehabilitation[Title/Abstract])) OR (Exercises, Rehabilitation[Title/Abstract])) OR (Rehabilitation Exercises[Title/Abstract])))) OR ((Exercise[MeSH Terms]) OR ((((((((((((((((((((((((Exercises[Title/Abstract]) OR (Physical Activity[Title/Abstract])) OR (Activities, Physical[Title/Abstract])) OR (Activity, Physical[Title/Abstrac])) OR (Physical Activities[Title/Abstract])) OR (Exercise, Physical[Title/Abstract])) OR (Exercises, Physical[Title/Abstract])) OR (Physical Exercise[Title/Abstract])) OR (Physical Exercises[Title/Abstract])) OR (Acute Exercise[Title/Abstract])) OR (Acute Exercises[Title/Abstract])) OR (Exercise, Acute[Title/Abstract])) OR (Exercises, Acute[Title/Abstract])) OR (Exercise, Isometric[Title/Abstract])) OR (Exercises, Isometric[Title/Abstract])) OR (Isometric Exercises[Title/Abstract])) OR (Isometric Exercise[Title/Abstract])) OR (Exercise, Aerobic[Title/Abstract])) OR (Exercise, Aerobic[Title/Abstract])) OR (Aerobic Exercise[Title/Abstract])) OR (Aerobic Exercises[Title/Abstract])) OR (Exercises, Aerobic[Title/Abstract])) OR (Exercise Training[Title/Abstract])) OR (Trainings, Exercise[Title/Abstract])))) OR (((((((((((((((((((((((((((((((((((((((((((Baseball[Title/Abstract]) OR (Basketball[Title/Abstract])) OR (Bicycling[Title/Abstract])) OR (Boxing[Title/Abstract])) OR (Cricket Sport[Title/Abstract])) OR (Football[Title/Abstract])) OR (Golf[Title/Abstract])) OR (Gymnastics[Title/Abstract])) OR (Hockey[Title/Abstract])) OR (Martial Arts[Title/Abstract])) OR (Mountaineering[Title/Abstract])) OR (Racquet[Title/Abstract])) OR (Return[Title/Abstract])) OR (Sport[Title/Abstract])) OR (Sports[Title/Abstract])) OR (Rugby[Title/Abstract])) OR (Running[Title/Abstract])) OR (Skating[Title/Abstract])) OR (Snow Sports[Title/Abstract])) OR (Soccer[Title/Abstract])) OR (train[Title/Abstract])) OR (fitness[Title/Abstract])) OR (aerobic[Title/Abstract])) OR (walking[Title/Abstract])) OR (high intensity interval[Title/Abstract])) OR (resistance[Title/Abstract])) OR (core stability[Title/Abstract])) OR (dance[Title/Abstract])) OR (breathing exercise[Title/Abstract])) OR (virtual reality exercise[Title/Abstract])) OR (whole body vibration exercise[Title/Abstract])) OR (stretching[Title/Abstract])) OR (body ⁃ mind exercise[Title/Abstract])) OR (Yoga[Title/Abstract])) OR (pilates[Title/Abstract])) OR (Tai Chi[Title/Abstract])) OR (Taijiquan[Title/Abstract])) OR (Health Qigong[Title/Abstract])) OR (Yijinjing[Title/Abstract])) OR (Wuqinxi[Title/Abstract])) OR (Liuzijue[Title/Abstract])) OR (Baduanjin[Title/Abstract])) OR (multicomponent exercise[Title/Abstract]))

#4：#1 AND #2 AND #3

WOS（299）

#1：((((TS=(Autism Spectrum Disorder)) OR TS=(Autism Spectrum Disorders)) OR TS=(Autistic Spectrum Disorder)) OR TS=(Autistic Spectrum Disorders)) OR TS=(Disorder, Autistic Spectrum)

#2：(TS=(Equine-Assisted Therapy)) OR (TS=(Animal Assisted Therapy)) OR (TS=(Baseball)) OR (TS=(Basketball)) OR (TS=(Bicycling)) OR (TS=(Boxing)) OR (TS=(Cricket Sport)) OR (TS=(Football)) OR (TS=(Golf)) OR (TS=(Gymnastics)) OR (TS=(Hockey)) OR (TS=(Martial Arts)) OR (TS=(Mountaineering)) OR (TS=(Racquet Sports)) OR (TS=(Return to Sport)) OR (TS=(Rugby)) OR (TS=(Running)) OR (TS=(Skating)) OR (TS=(Snow Sports)) OR (TS=(Soccer)) OR (TS=(train)) OR (TS=(fitness)) OR (TS=(aerobic)) OR (TS=(walking)) OR (TS=(high intensity interval)) OR (TS=(resistance)) OR (TS=(core stability)) OR (TS=(dance)) OR (TS=(breathing exercise)) OR (TS=(virtual reality exercise)) OR (TS=(whole body vibration exercise)) OR (TS=(stretching)) OR (TS=(body ⁃ mind exercise)) OR (TS=(Yoga)) OR (TS=(pilates)) OR (TS=(Tai Chi)) OR (TS=(Taijiquan)) OR (TS=(Health Qigong)) OR (TS=(Yijinjing)) OR (TS=(Wuqinxi)) OR (TS=(Liuzijue)) OR (TS=(Baduanjin)) OR (TS=(multicomponent exercise)) OR (((TS=(Sports)) OR TS=(Sport)) OR TS=(Athletics)) OR TS=(Athletic)OR (((((((((((TS=(Exercise Therapy)) OR TS=(Remedial Exercise)) OR TS=(Exercise, Remedial)) OR TS=(Exercises, Remedial)) OR TS=(Remedial Exercises)) OR TS=(Therapy, Exercise)) OR TS=(Exercise Therapies)) OR TS=(Therapies, Exercise)) OR TS=(Rehabilitation Exercise)) OR TS=(Exercise, Rehabilitation)) OR TS=(Exercises, Rehabilitation)) OR TS=(Rehabilitation Exercises)

OR (((((((((((((((((((((((((TS=(Exercise)) OR TS=(Exercises)) OR TS=(Physical Activity)) OR TS=(Activities, Physical)) OR TS=(Activity, Physical)) OR TS=(Physical Activities)) OR TS=(Exercise, Physical)) OR TS=(Exercises, Physical)) OR TS=(Physical Exercise)) OR TS=(Physical Exercises)) OR TS=( Acute Exercise)) OR TS=(Acute Exercises)) OR TS=(Exercise, Acute)) OR TS=(Exercises, Acute)) OR TS=(Exercise, Isometric)) OR TS=(Exercises, Isometric)) OR TS=(Isometric Exercises)) OR TS=(Isometric Exercise)) OR TS=(Exercise, Aerobic)) OR TS=(Aerobic Exercise)) OR TS=(Aerobic Exercises)) OR TS=(Exercises, Aerobic)) OR TS=(Exercise Training)) OR TS=(Exercise Trainings)) OR TS=(Training, Exercise)) OR TS=(Trainings, Exercise)

#3：(((((TS=(Executive Functions)) OR TS=(Executive Functions)) OR TS=(Function, Executive)) OR TS=(Functions, Executive)) OR TS=(Executive Control)) OR TS=(Executive Controls)

#: (((((TS=(Executive Functions)) OR TS=(Executive Functions)) OR TS=(Function, Executive)) OR TS=(Functions, Executive)) OR TS=(Executive Control)) OR TS=(Executive Controls)

#4：#1 AND #2 AND #3

'autism spectrum disorders':ab,ti OR 'autistic spectrum disorder':ab,ti OR 'autistic spectrum disorders':ab,ti OR 'disorder, autistic spectrum':ab,ti

Embase（463）

#1：'sport':ab,ti OR 'athletics':ab,ti OR 'athletic':ab,ti OR 'remedial exercise':ab,ti OR 'exercise, remedial':ab,ti OR 'exercises, remedial':ab,ti OR 'remedial exercises':ab,ti OR 'therapy, exercise':ab,ti OR 'exercise therapies':ab,ti OR 'therapies, exercise':ab,ti OR 'rehabilitation exercise':ab,ti OR 'exercise, rehabilitation':ab,ti OR 'exercises, rehabilitation':ab,ti OR 'rehabilitation exercises':ab,ti OR 'exercises':ab,ti OR 'physical activity':ab,ti OR 'activities, physical':ab,ti OR 'activity, physical':ab,ti OR 'physical activities':ab,ti OR 'exercise, physical':ab,ti OR 'exercises, physical':ab,ti OR 'physical exercise':ab,ti OR 'physical exercises':ab,ti OR 'acute exercise':ab,ti OR 'acute exercises':ab,ti OR 'exercise, acute':ab,ti OR 'exercises, acute':ab,ti OR 'exercise, isometric':ab,ti OR 'exercises, isometric':ab,ti OR 'isometric exercises':ab,ti OR 'isometric exercise':ab,ti OR 'exercise, aerobic':ab,ti OR 'aerobic exercise':ab,ti OR 'aerobic exercises':ab,ti OR 'exercises, aerobic':ab,ti OR 'exercise training':ab,ti OR 'exercise trainings':ab,ti OR 'training, exercise':ab,ti OR 'trainings, exercise':ab,ti OR 'baseball':ab,ti OR 'basketball':ab,ti OR 'bicycling':ab,ti OR 'boxing':ab,ti OR 'cricket sport':ab,ti OR 'football':ab,ti OR 'golf':ab,ti OR 'gymnastics':ab,ti OR 'hockey':ab,ti OR 'martial arts':ab,ti OR 'mountaineering':ab,ti OR 'racquet sports':ab,ti OR 'return to sport':ab,ti OR 'rugby':ab,ti OR 'running':ab,ti OR 'skating':ab,ti OR 'snow sports':ab,ti OR 'soccer':ab,ti OR 'train':ab,ti OR 'fitness':ab,ti OR 'aerobic':ab,ti OR 'walking':ab,ti OR 'high intensity interval':ab,ti OR 'resistance':ab,ti OR 'core stability':ab,ti OR 'dance':ab,ti OR 'breathing exercise':ab,ti OR 'virtual reality exercise':ab,ti OR 'whole body vibration exercise':ab,ti OR 'stretching':ab,ti OR 'body mind exercise':ab,ti OR 'yoga':ab,ti OR 'pilates':ab,ti OR 'tai chi':ab,ti OR 'taijiquan':ab,ti OR 'health qigong':ab,ti OR 'yijinjing':ab,ti OR 'wuqinxi':ab,ti OR 'liuzijue':ab,ti OR 'baduanjin':ab,ti OR 'multicomponent exercise':ab,ti OR 'equine-assisted therapy':ab,ti OR 'animal assisted therapy':ab,ti

#2： 'autism spectrum disorders':ab,ti OR 'autistic spectrum disorder':ab,ti OR 'autistic spectrum disorders':ab,ti OR 'disorder, autistic spectrum':ab,ti

#3：#1 AND #2

Cochrane（）

#1：(Sport ):ab,ti,kw OR (Athletics ):ab,ti,kw OR (Athletic ):ab,ti,kw OR (Remedial Exercise ):ab,ti,kw OR (Exercise, Remedial ):ab,ti,kw OR (Exercises, Remedial ):ab,ti,kw OR (Remedial Exercises ):ab,ti,kw OR (Therapy, Exercise ):ab,ti,kw OR (Exercise Therapies ):ab,ti,kw OR (Therapies, Exercise ):ab,ti,kw OR (Rehabilitation Exercise ):ab,ti,kw OR (Exercise, Rehabilitation ):ab,ti,kw OR (Exercises, Rehabilitation ):ab,ti,kw OR (Rehabilitation Exercises ):ab,ti,kw OR (Exercises ):ab,ti,kw OR (Physical Activity ):ab,ti,kw OR (Activities, Physical ):ab,ti,kw OR (Activity, Physical ):ab,ti,kw OR (Physical Activities ):ab,ti,kw OR (Exercise, Physical ):ab,ti,kw OR (Exercises, Physical ):ab,ti,kw OR (Physical Exercise ):ab,ti,kw OR (Physical Exercises ):ab,ti,kw OR (Acute Exercise ):ab,ti,kw OR (Acute Exercises ):ab,ti,kw OR (Exercise, Acute ):ab,ti,kw OR (Exercises, Acute ):ab,ti,kw OR (Exercise, Isometric ):ab,ti,kw OR (Exercises, Isometric ):ab,ti,kw OR (Isometric Exercises ):ab,ti,kw OR (Isometric Exercise ):ab,ti,kw OR (Exercise, Aerobic ):ab,ti,kw OR (Aerobic Exercise ):ab,ti,kw OR (Aerobic Exercises ):ab,ti,kw OR (Exercises, Aerobic ):ab,ti,kw OR (Exercise Training ):ab,ti,kw OR (Exercise Trainings ):ab,ti,kw OR (Training, Exercise ):ab,ti,kw OR (Trainings, Exercise ):ab,ti,kw OR (Baseball ):ab,ti,kw OR (Basketball ):ab,ti,kw OR (Bicycling ):ab,ti,kw OR (Boxing ):ab,ti,kw OR (Cricket Sport ):ab,ti,kw OR (Football ):ab,ti,kw OR (Golf ):ab,ti,kw OR (Gymnastics ):ab,ti,kw OR (Hockey ):ab,ti,kw OR (Martial Arts ):ab,ti,kw OR (Mountaineering ):ab,ti,kw OR (Racquet Sports ):ab,ti,kw OR (Return to Sport ):ab,ti,kw OR (Rugby ):ab,ti,kw OR (Running ):ab,ti,kw OR (Skating ):ab,ti,kw OR (Snow Sports ):ab,ti,kw OR (Soccer ):ab,ti,kw OR (train ):ab,ti,kw OR (fitness ):ab,ti,kw OR (aerobic ):ab,ti,kw OR (walking ):ab,ti,kw OR (high intensity interval ):ab,ti,kw OR (resistance ):ab,ti,kw OR (core stability ):ab,ti,kw OR (dance ):ab,ti,kw OR (breathing exercise ):ab,ti,kw OR (virtual reality exercise ):ab,ti,kw OR (whole body vibration exercise ):ab,ti,kw OR (stretching ):ab,ti,kw OR (body mind exercise ):ab,ti,kw OR (Yoga ):ab,ti,kw OR (pilates ):ab,ti,kw OR (Tai Chi ):ab,ti,kw OR (Taijiquan ):ab,ti,kw OR (Health Qigong ):ab,ti,kw OR (Yijinjing ):ab,ti,kw OR (Wuqinxi ):ab,ti,kw OR (Liuzijue ):ab,ti,kw OR (Baduanjin ):ab,ti,kw OR (multicomponent exercise ):ab,ti,kw OR (Equine-Assisted Therapy ):ab,ti,kw OR (Animal Assisted Therapy ):ab,ti,kw

#2：(Autism Spectrum Disorders ):ab,ti,kw OR (Autistic Spectrum Disorder ):ab,ti,kw OR (Autistic Spectrum Disorders ):ab,ti,kw OR (Disorder, Autistic Spectrum ):ab,ti,kw

#3：#1 AND #2

## Regression analysis
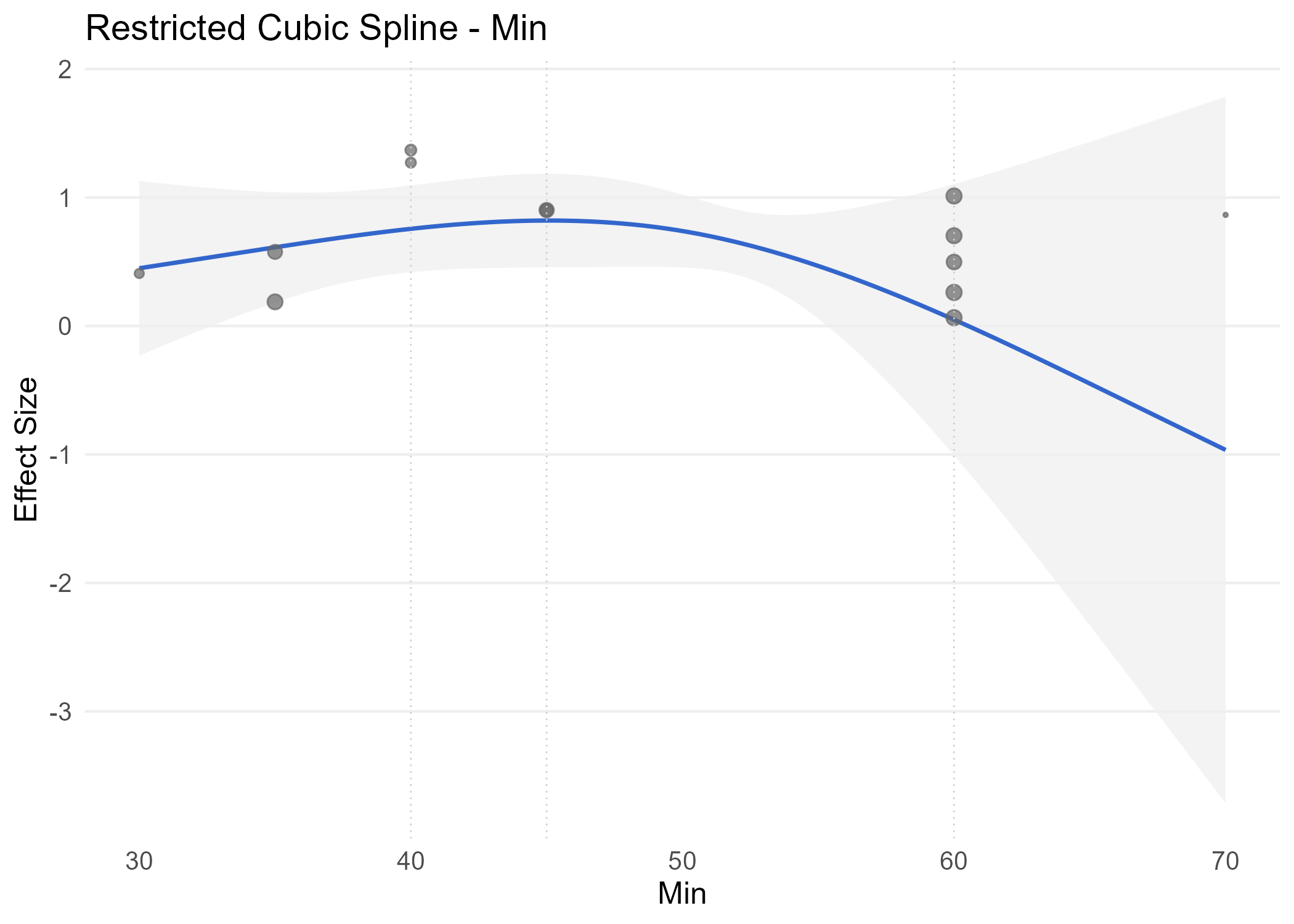

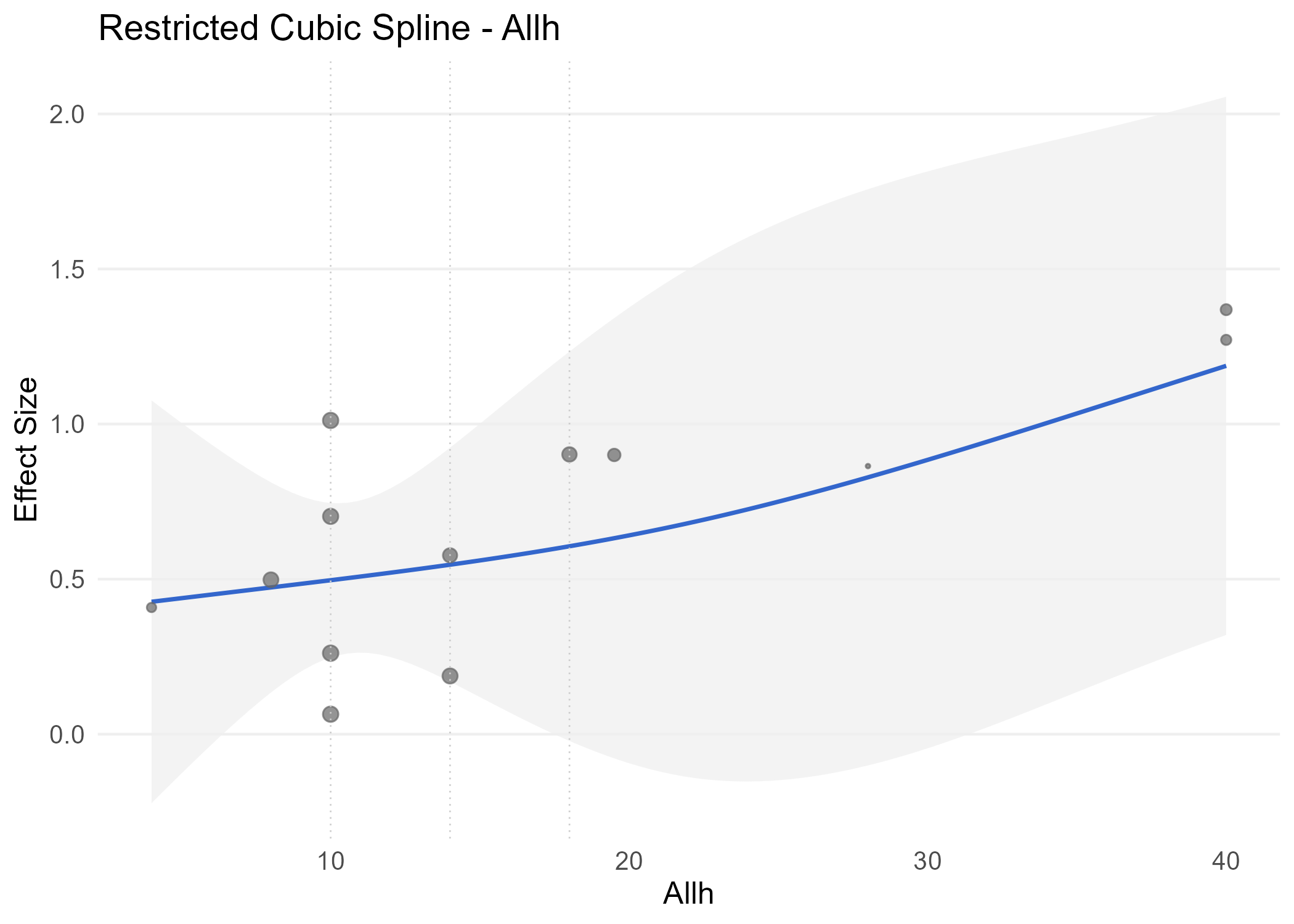

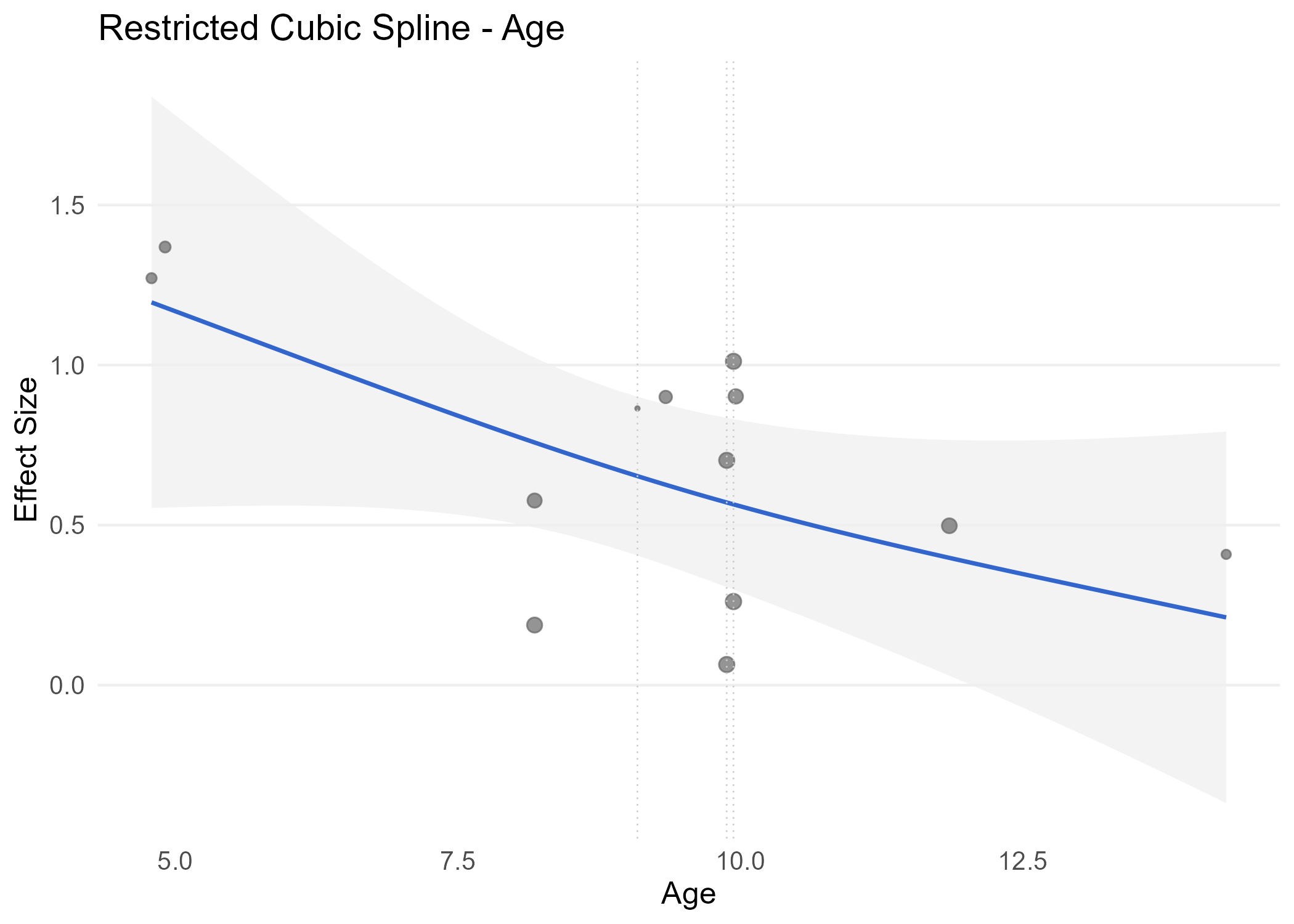

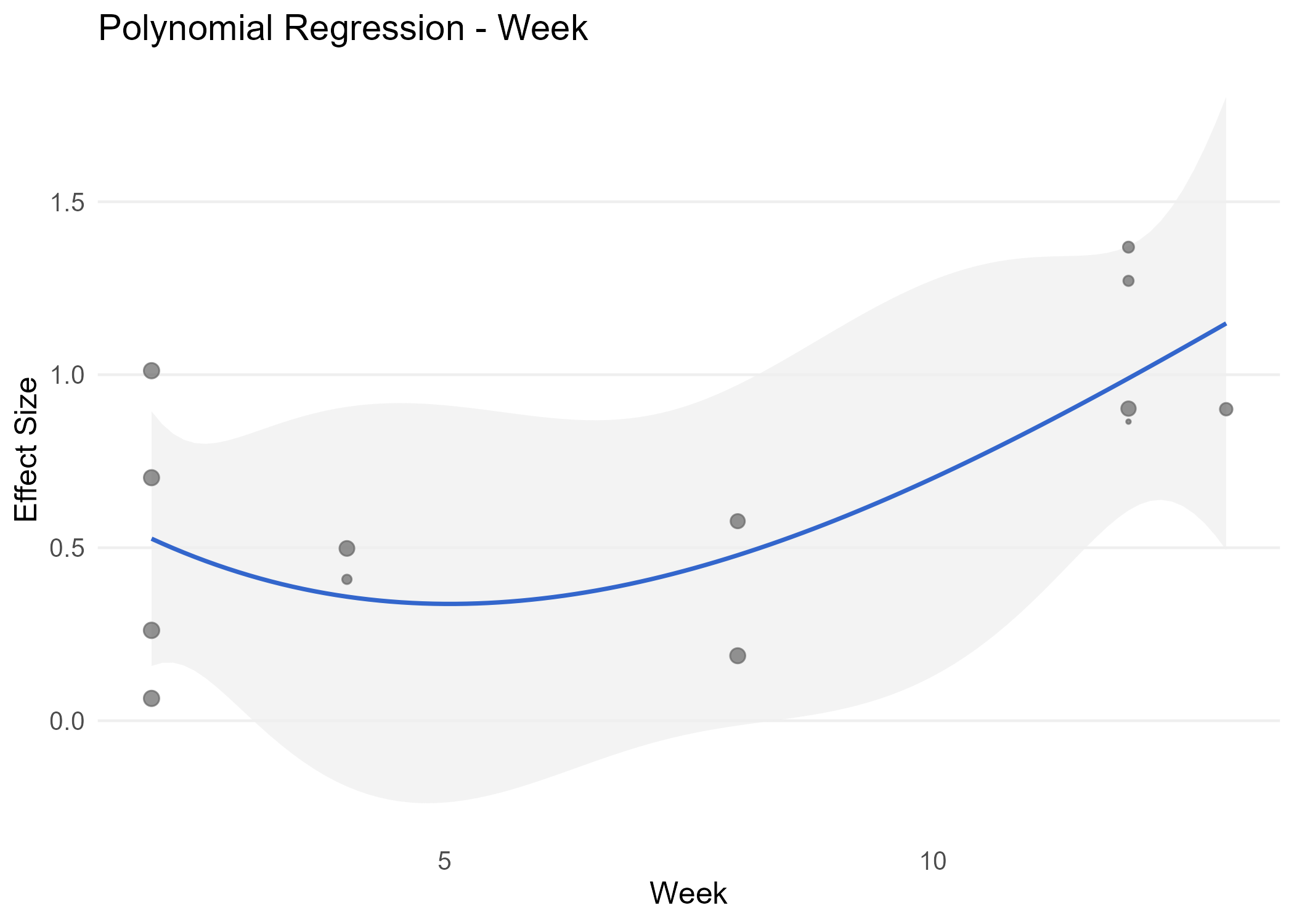

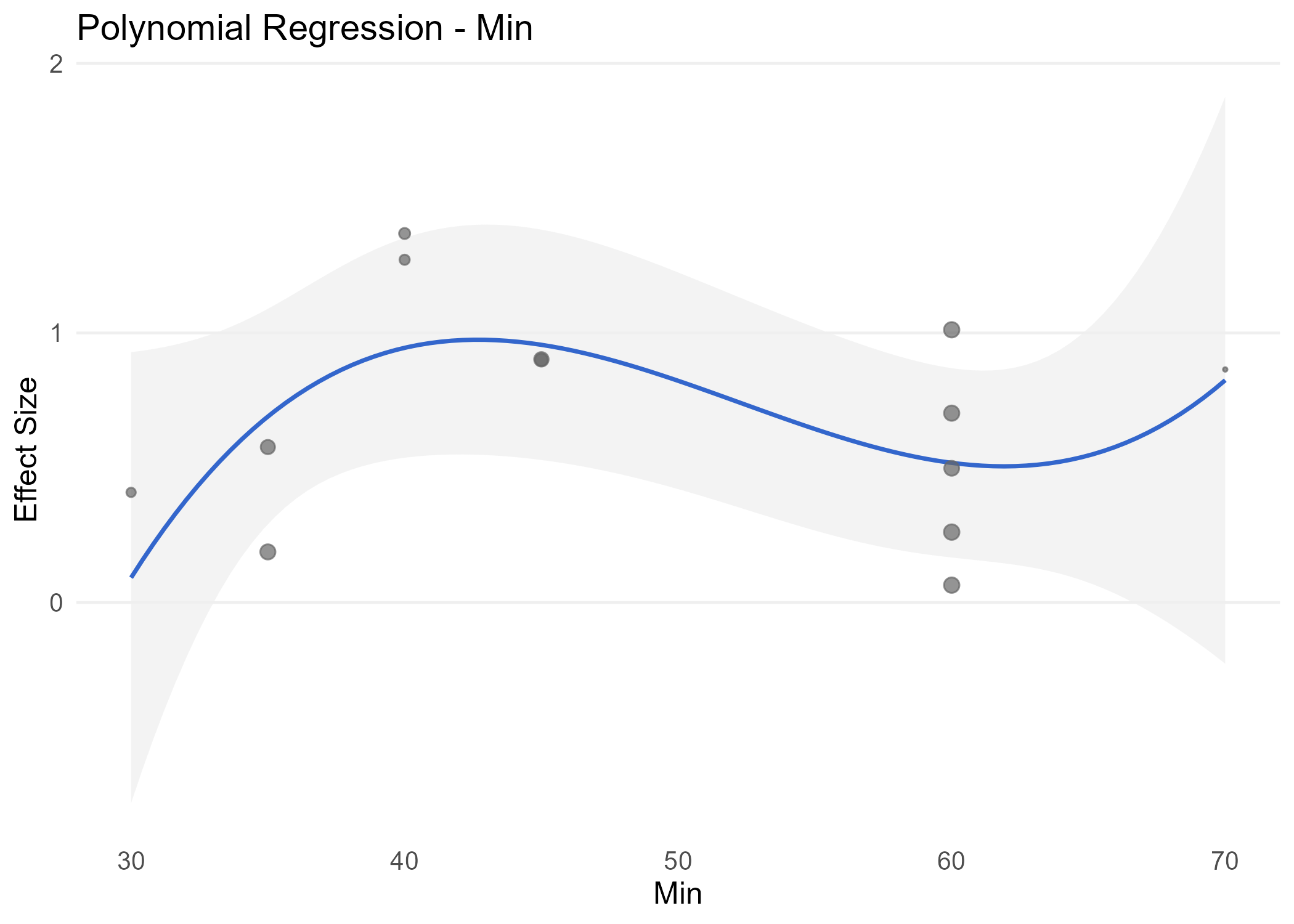

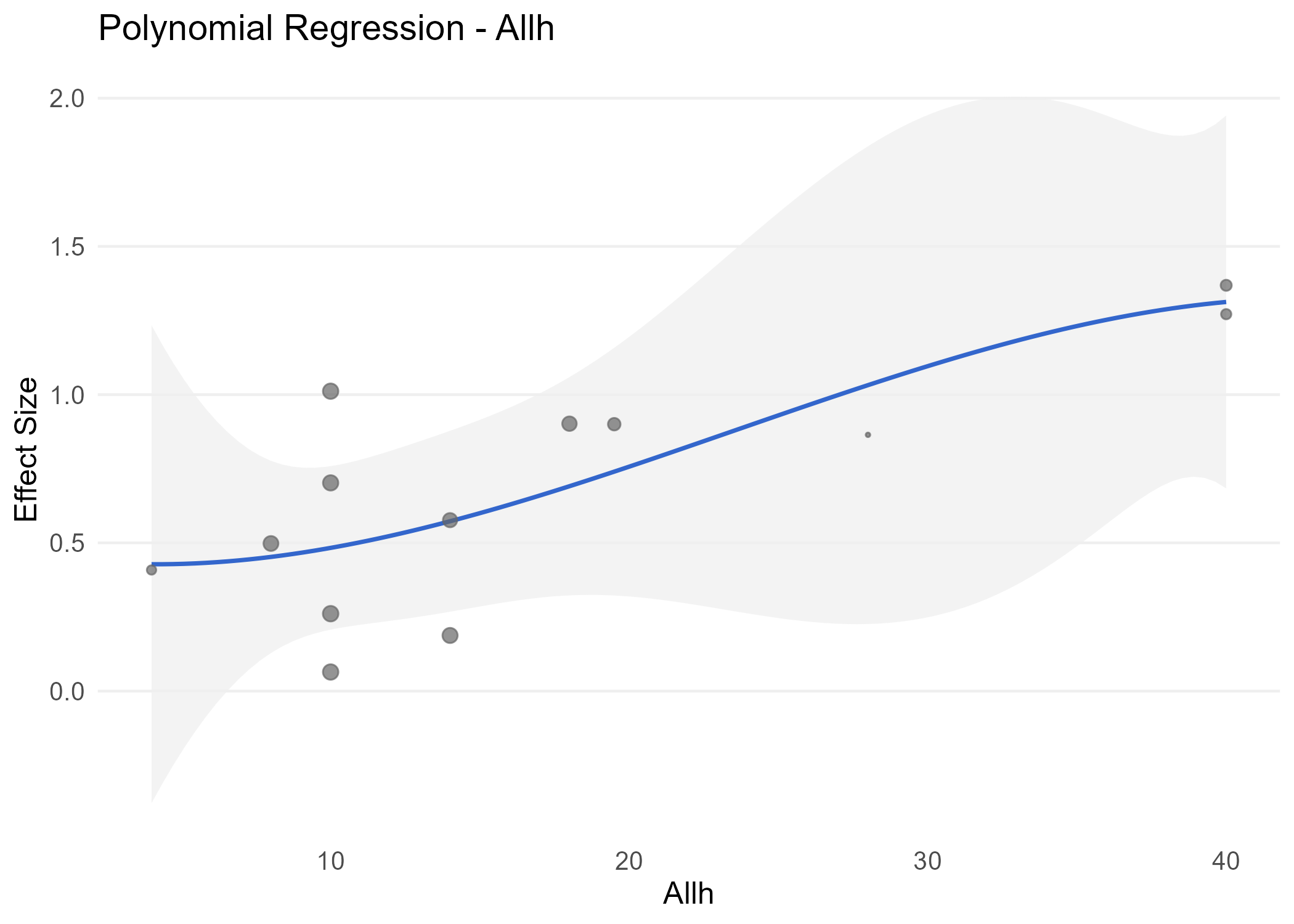

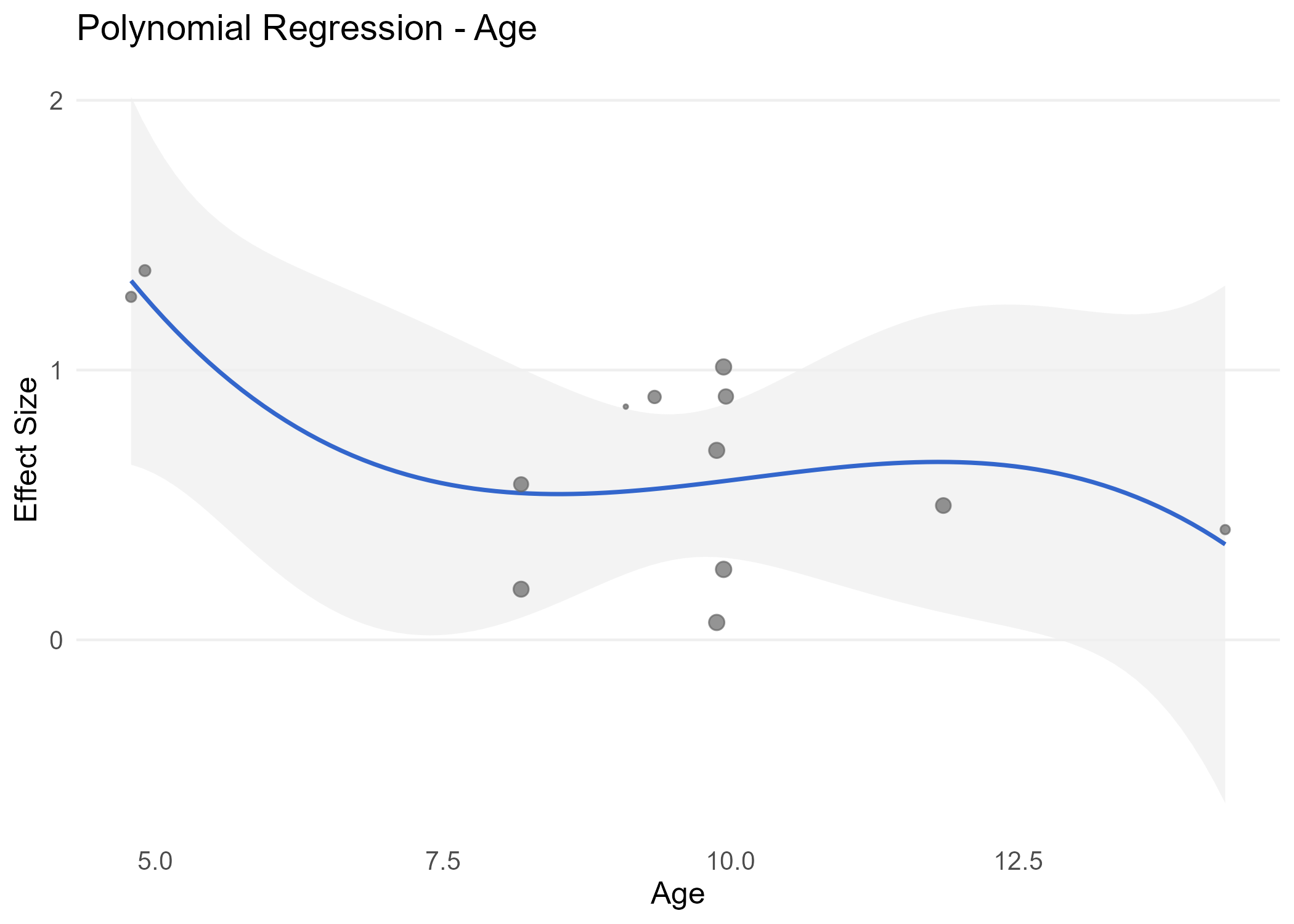

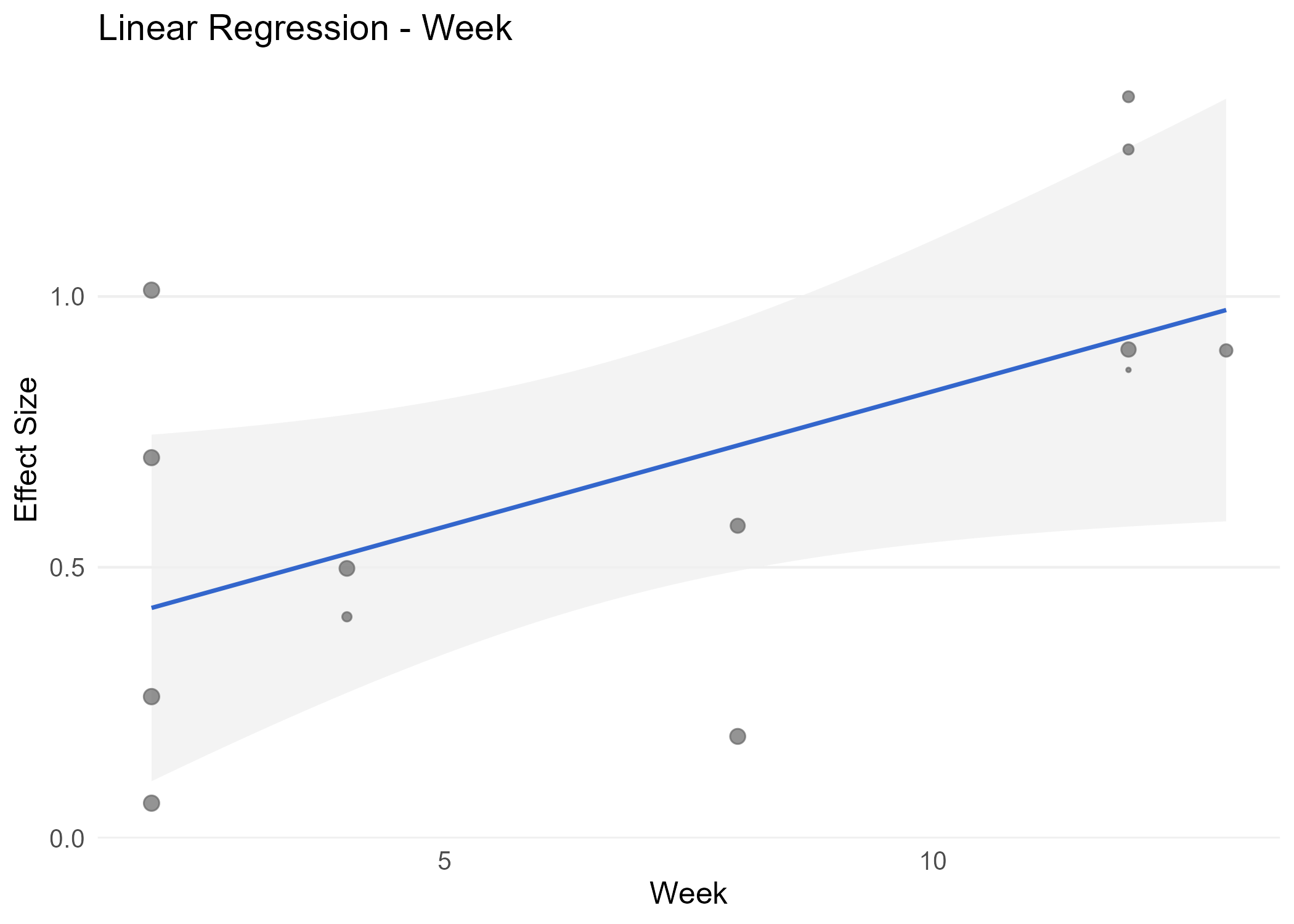

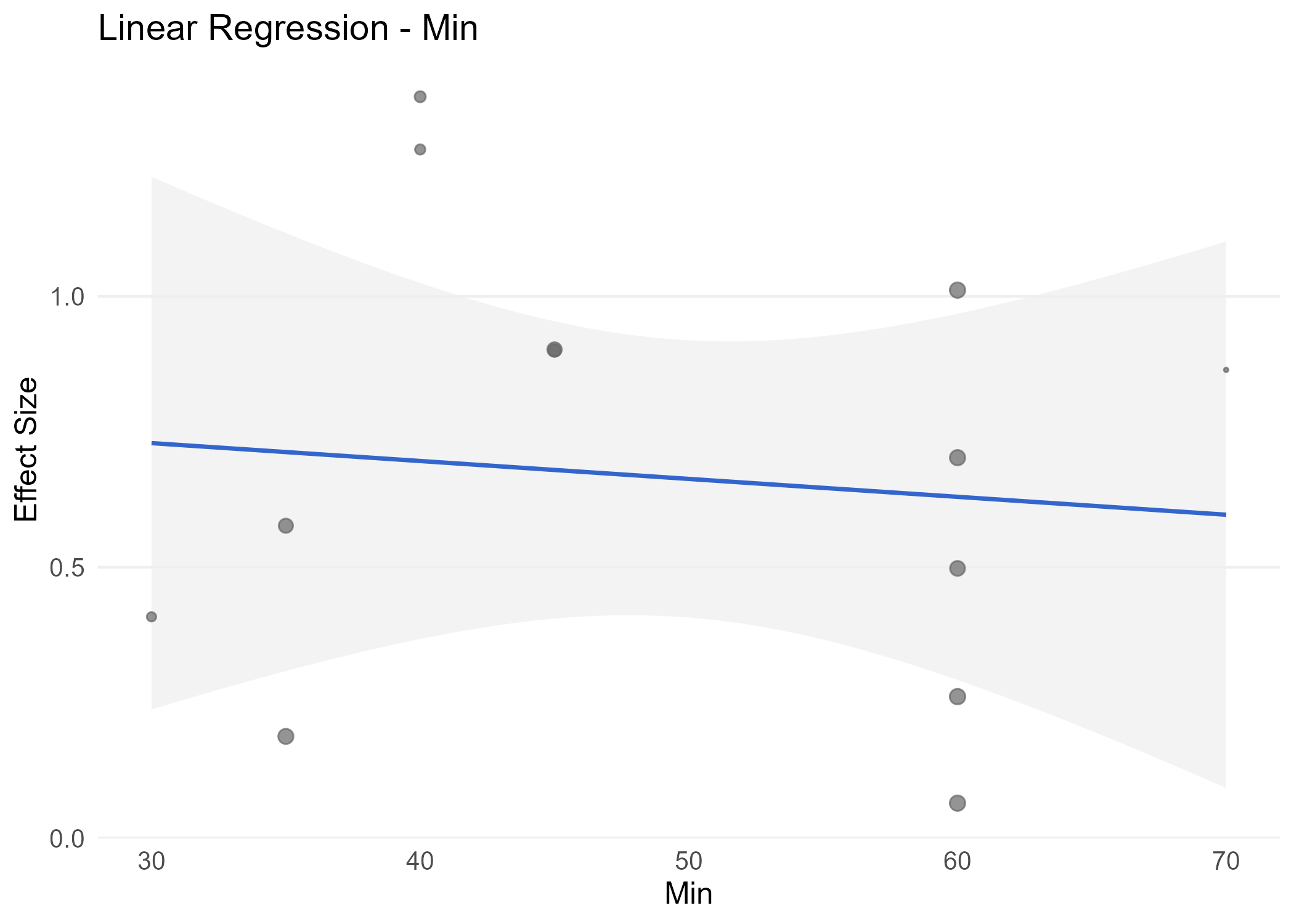

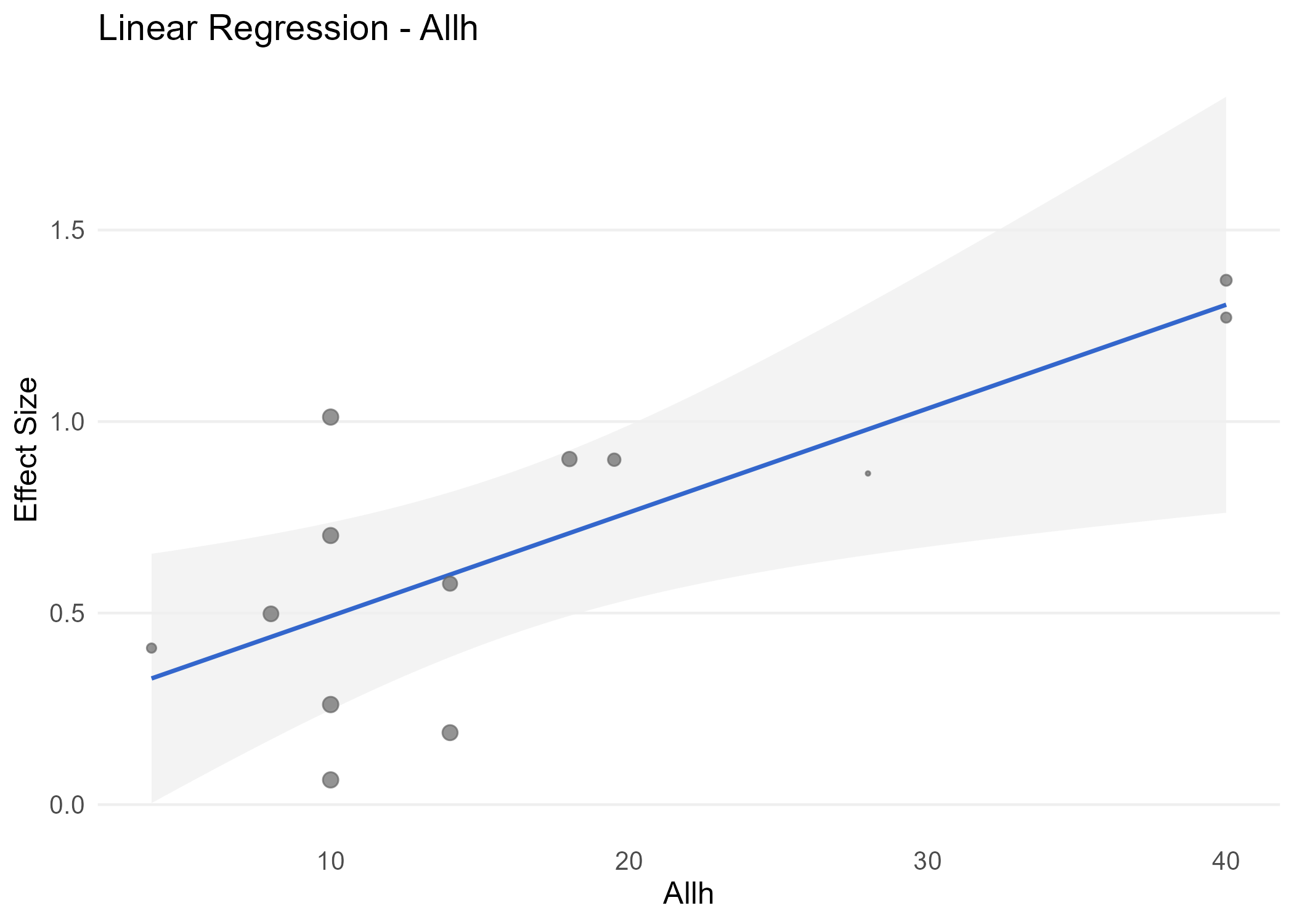

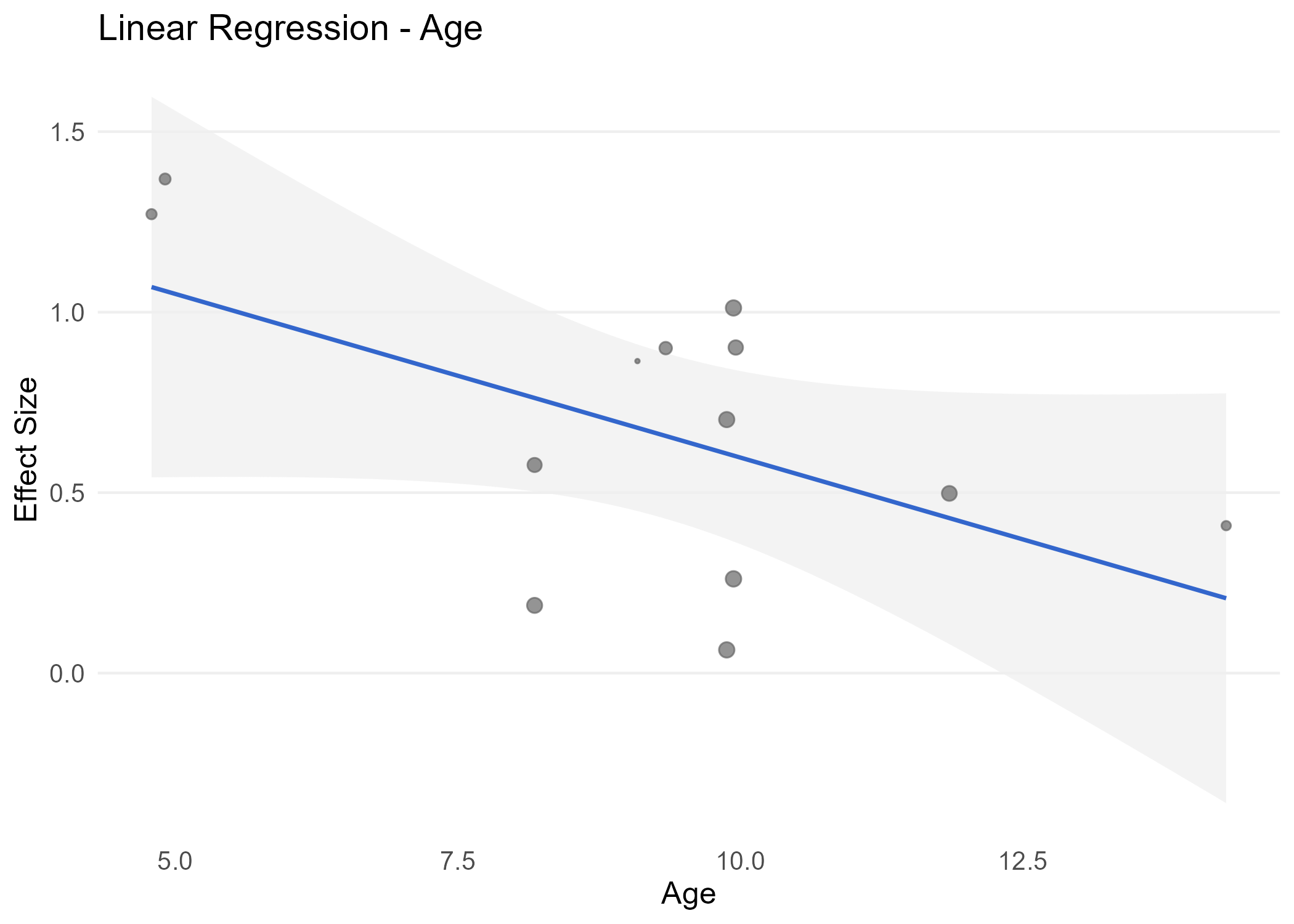


GRADE Evidence Certainty Assessment Table

| Assessment Domain | Rating | Reason for Downgrading/Upgrading |
| --- | --- | --- |
| Initial Certainty | High | All included studies were randomized or quasi-randomized controlled trials |
| Downgrading Factors |  |  |
| Risk of bias | -1 | 2 of 10 studies classified as high risk (Wang(50) and Liu(51)), 4 showed some concerns; significant issues in intervention adherence and blinding procedures implementation |
| Inconsistency of results | -1 | Heterogeneity in effect sizes between studies (SMDs ranging from 0.06 to 1.37); inconsistent results in control group outcomes with some showing negative changes (SMDs of -0.75) |
| Indirectness of evidence | No downgrade | All studies directly addressed target population (children and adolescents with ASD) and intervention (exercise interventions) |
| Imprecision of effect estimates | -1 | Small sample sizes in individual studies (11-23 participants per group); several subgroup interventions had confidence intervals crossing zero (CS, BR, SG, TT) |
| Publication bias | No downgrade | Funnel plot analysis showed marginal p-values (p=0.071 and p=0.062), but visual inspection did not reveal substantial asymmetry |
| Upgrading Factors |  |  |
| Large effect | No upgrade | Overall effect size is only moderate (SMD=0.66), does not meet criteria for large effect |
| Dose-response gradient | +1 | Total training duration showed significant positive correlation with effect size (β=0.027, p=0.015) |
| Potential confounding factors | No upgrade | Insufficient evidence that confounding factors would reduce observed effect |
| Final Certainty Rating | Low | Limitations in study design, inconsistency between studies, and imprecision in effect estimates reduce confidence in the findings |

Evidence Assessment Explanation

Low certainty evidence indicates: - We have limited confidence in the effect estimate for exercise interventions on inhibitory control in children with ASD - The true effect may be substantially different from the estimated effect - Major limitations include: - Methodological issues in blinding and intervention adherence - Substantial variation in effect sizes across studies - Small sample sizes (ranging from 11-23 participants per group) - Several intervention types did not reach statistical significance - While total training duration shows a positive correlation with outcomes, the overall evidence quality is compromised by study limitations - Future research is likely to have an important impact on confidence in the estimate of effect and may change the estimate
